# Engineering Host–Guest Interactions for Next-Generation Heparin Biosensors

**DOI:** 10.1002/smll.74588

**Published:** 2026-07-29

**Authors:** Frank Boateng Osei, Charles Mariasoosai, Zackary Christodolu, Chloe Caringi, Sahar Heidari, Kwaku Twum, Sithara Nanjappa, Evan Trivedi, A. Valance Washington, Hedieh Torabifard, Ngong Kodiah Beyeh

**Affiliations:** 1 Department of Chemistry, Oakland University, Rochester, Michigan, USA; 2 Department of Chemistry and Biochemistry, The University of Texas at Dallas, Richardson, Texas, USA; 3 Department of Biological Sciences, Oakland University, Rochester, Michigan, USA; 4 Department of Pathology and Laboratory Medicine, Boston University School of Medicine, Boston, Massachusetts, USA

**Keywords:** heparin neutralization, heparin sensing, indicator displacement assay, noncovalent interactions, pillararenes

## Abstract

Heparin, a polyanionic anticoagulant, is widely used clinically but requires neutralization to restore normal blood coagulation. Protamine sulfate (**PS**) is the standard antidote, yet limitations exist. Herein, a polycationic, cavity-containing pillar[6]arene macrocycle (**P12+**) demonstrates superior heparin neutralization in physiological media compared to its lower-valency analog **P10+**, and performance comparable to **PS**. MTT assays reveal low toxicity of the **P12+**, suggesting feasibility for in vivo use. Dynamic light scattering reveals that **P12+** forms nanoscale complexes below 400 nm, smaller than **PS**-heparin aggregates. Remarkably, **P12+** exhibits enhanced neutralization of low-molecular-weight heparin, a known limitation of **PS**. Additionally, heparin detection is achieved via a host–guest indicator displacement assay (IDA) using methyl orange (**MO**). UV–vis studies show ~80% **MO** release with **P12+**, compared to ~40% with **P10+** in the presence of heparin. This response is detectable in undiluted human plasma for **P12+**, whereas **P10+** shows a suitable response in 5% diluted plasma. A fluorescence-based IDA enables sensitive detection down to 0.11 U/mL, below the therapeutic range (0.80–2.00 U/mL). Molecular dynamics simulations support these findings, revealing that weaker host–guest interactions and favorable electrostatics in **P12+** facilitate efficient **MO** release.

## 1 ∣ Introduction

Heparin, a sulfated anionic polysaccharide belonging to the group of glycosaminoglycans, is commonly used as an anticoagulant in clinical settings [1-3]. The anticoagulant effect is achieved through activation of antithrombin-III, which in turn, inactivates thrombin and factor Xa to prevent fibrin and clot formation [4-6]. Heparin antidotes are often necessary to balance and neutralize heparin dosing to prevent excessive bleeding with heparin use [7-10]. Protamine sulfate (**PS**), an arginine-rich cationic polypeptide, is a commonly used heparin neutralizer in clinical settings [11-14]. This neutralization is achieved mainly via electrostatic interactions between the anionic heparin and cationic **PS** [11, 15, 16]. **PS**’s interaction with heparin is, however, non-specific, and its use is marked with major adverse effects such as allergic reactions, bradycardia, systemic hypotension, pulmonary vasoconstriction, and recent reports of liver and kidney damage [13, 17-19]. This neutralizer has been found to cause paradoxical anticoagulation effect and is less effective against low molecular weight heparins [20-22]. Safer and effective alternatives for heparin neutralization would be important to avoid such effects from **PS**. Various biological and chemical models have been investigated for their ability to neutralize heparin. Biological models studied for heparin neutralization include engineered protein cages [23, 24], protein copolymers [25], albumin-peptide conjugates [26], and a cartridge-based heparinase system [27]. Limitations of such models include difficulty in design and characterization [28]. Some chemical-based heparin neutralizers include a poly(ethylene glycol)-poly(2-(dimethylamino)ethyl methacrylate block copolymers [29], quantum dots [30, 31], small molecule probes [32], and bis-2-methyl-4-amino-quinolyl-6-carbamide (surfen) [33] for heparin neutralization. In addition, cationic calix[8]arenes, resorcinarenes, and pillar[5]arene macrocycles have been studied for potential heparin neutralization via electrostatic interactions [3, 34]. Polyionic pillar[n]arenes as heparin neutralizers would afford highly soluble, generally safe, and relatively cheaper alternatives. Their neutralizing capacity depends on their polyionic charge density. We recently reported on a polycationic cavity-containing pillar[5]arene, **P10+**, as a superior heparin neutralizer compared to several polycationic resorcinarenes (with charge densities of 4+, 8+, and 16+). This increase in heparin neutralizing ability was attributed to **P10+** bearing charge density on both rims, whereas the polycationic resorcinarenes possess cationic groups only on their upper rims. Multivalency and accessibility are both responsible for **P10+**’s heparin-neutralizing ability [3].

Heparin detection is essential for monitoring heparin concentrations even after administration of a neutralizer [35-37]. Traditional methods, which include activated partial thromboplastin time (aPTT) and activated clotting time (ACT) assays, are indirect, less sensitive, less selective, and have been reported to be less accurate [38-41]. The search for improved heparin detectors, many of which are based on colorimetric and fluorescent sensing methods, is ongoing. A variety of these detectors are based on quantum dots [30, 31], conjugated protein polymers [29], and small molecule probes [32]. Apart from the difficulty and cost involved in synthesizing some of these models in large quantities, another key limitation is their ability to detect heparin in higher concentrations of serum or plasma [38, 42]. A highly efficient heparin sensor should permit detection in high proportions of serum (50% and above) [38, 42], which can be potentiated by increasing the charge density on the probe. Cavity-containing **P10+** macrocycle, with 10 positive charges, has been studied as an ultraviolet and visible (UV–vis) absorption-based heparin detector via indicator displacement assay (IDA) with methyl orange (**MO**) dye. However, in the report, only 40% release of the assay indicator was determined, and could foster a suitable detection calibration curve in only 5% Tris buffered diluted human plasma. This is attributed to a stronger binding affinity between the **P10+** and the **MO** dye, thus limiting the release of **MO** in the presence of heparin [3].

**FIGURE 1 ∣:**
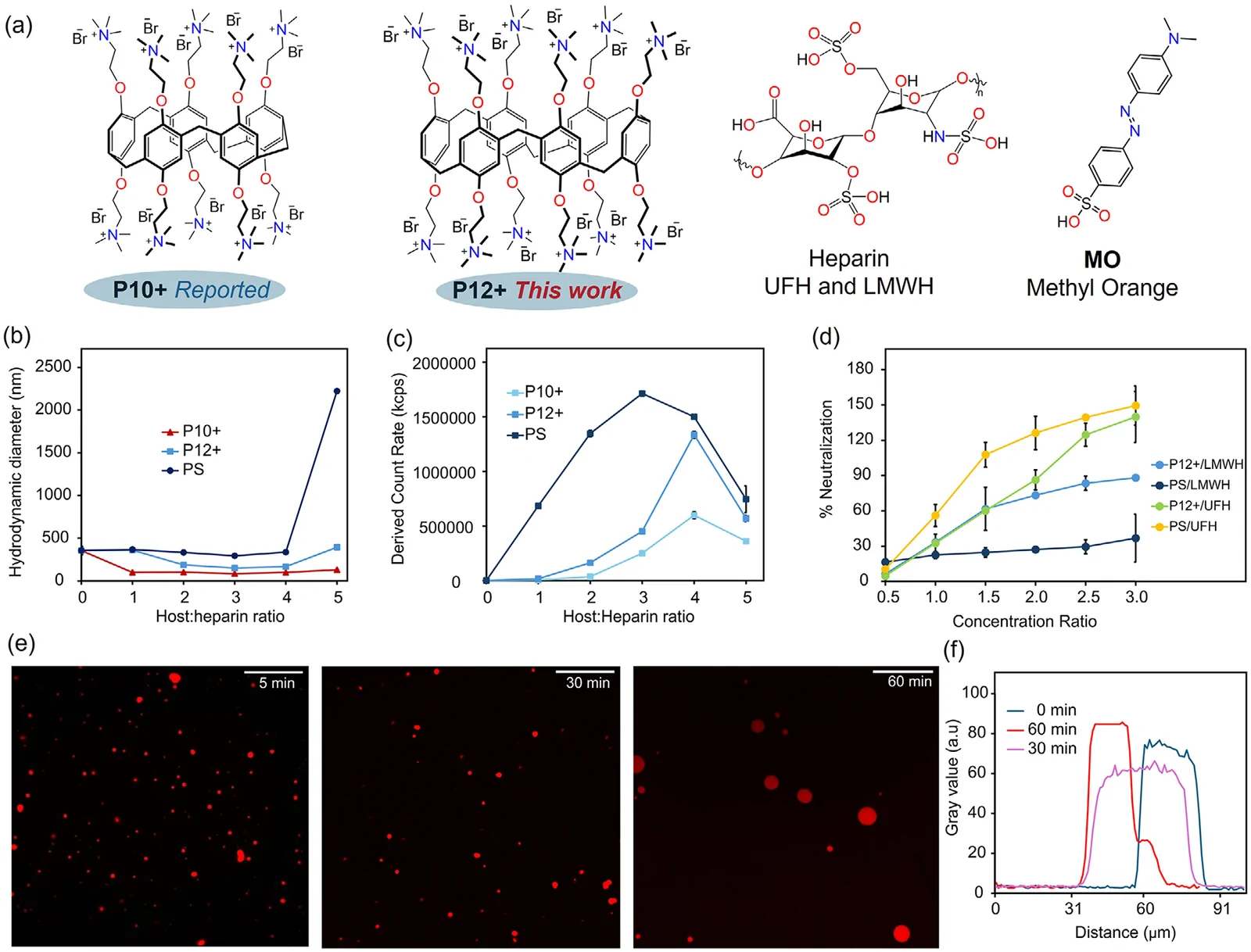
The components and complexation between the polyionic macrocycles (**P10+** and **P12+**) and heparin were studied in a physiologically relevant Tris-NaCl buffer (10 mM Tris, 150 mM NaCl, pH 7.4). **P12+**/heparin complex formation was studied using dynamic light scattering (DLS) and compared with complexes with **P10+** and **PS**. (a) The components. (b) The hydrodynamic diameter of heparin/binder complexes and (c) the derived count rate. The heparin neutralizing ability of **P12+** was studied using confocal fluorescence microscopy (CFM) and the chromogenic anti-Xa assay in plasma. (d) The anti-Xa assay data of neutralizers (**P12+** and **PS**) against unfractionated (UFH) and low-molecular-weight heparins (LMWH). (e) CFM images of the time-dependent scavenging ability of **P12+** on a Rhodamine-B-labelled heparin at 595 nm. (f) Line graphs of the time-dependent heparin scavenging effect of **P12+**. All experiments were done in triplicate. Data represent means ± standard error of the mean (S.E.M.). Some of the standard deviations are too small to be discernible.

Herein, we hypothesize that a larger pillar[6]arene **P12+** macrocycle possessing more cationic charge density could be a more effective heparin neutralizer than the previously reported **P10+** (Figure 1a). Furthermore, **P12+**’s larger cavity is expected to facilitate improved release of the **MO** indicator, thereby enabling a more efficient sensor for heparin. Accordingly, the **P12+** macrocycle represents a significant advancement over the previously reported **P10+** system, exhibiting enhanced heparin neutralization and detection in physiological media and undiluted human plasma. This dual functionality positions **P12+** as a uniquely powerful platform with both therapeutic and diagnostic potential. This process was investigated through a series of analytical procedures such as calorimetry, NMR, UV–vis, fluorescence spectroscopy, and microscopy. MTT dye and hemolytic assays were done to assess the cytotoxicity of the macrocycle. To complement our experimental findings, we conducted molecular dynamics (MD) simulations to investigate the interactions between the **MO** indicator and **P12+/P10+** and the flexibility of **MO** within these complexes. Results show weaker interactions between **P12+** and **MO**, and greater flexibility of **MO** compared to **P10+**. Steered molecular dynamics (SMD) simulations with a heparin octamer reveal the mechanism of **MO** ejection from the macrocycle’s cavity, in which cationic groups at the rims facilitate its egress.

## 2 ∣ Results and Discussion

### 2.1 ∣ Heparin Scavenging and Neutralization

Here, we investigated the potential of using pillar[6]arene macrocycle **P12+** as: (a) a heparin scavenger/neutralizer and (b) a heparin detector. First, the effective heparin-binding activity of the macrocycles and **PS** was confirmed with changes in their hydrodynamic diameters from DLS measurements upon the addition of increasing amounts of heparin. The macrocycles **P10+** and **P12+** formed uniform complexes with heparin up to particle sizes of 129 and 393 nm, respectively, while **PS** forms complexes of up to 2 μm with Heparin (Figure 1b). The derived count rate, an indicator of light scattering ability and complex formation, was also determined. Larger count rates indicate more complexes. At the same mass ratios, **PS** formed more complexes while **P10+** formed the least (Figure 1c and Figures S7-S9). The difference could be attributed to their respective charge density available for electrostatic interactions with heparin.

Further, we performed isothermal titration calorimetry (ITC) experiments between heparin and the macrocycles **P10+** and **P12+** to obtain thermodynamic parameters of the interactions. The dissociation constant (*K*_d_) shows a stronger interaction between **P12+** and heparin as compared to **P10+**. The data show a spontaneous binding process driven by entropy for both macrocycles and heparin (Table 1 and Figure S5). With the improved binding of **P12+** over **P10+** towards heparin, we then tested its neutralizing potential towards low-molecular-weight (LMW) and unfractionated (UFH) heparins under physiological conditions (Tris-buffered saline, pH 7.40) via Anti-Xa assay. Results indicate a stronger neutralization effect towards UFH than LMW heparins, which is due to the lower anionic charge density on the latter necessary for electrostatic interactions (Figure 1d and Figure S10). **PS** is known to weakly neutralize LMW heparins due to the smaller size of the heparin and fewer sulfate groups compared to UFH [13, 43, 44]. This is evident in our findings, as it shows weaker neutralization against LMW heparin. Interestingly, the **P12+** show a stronger neutralization effect with LMW heparin as compared to **PS** (Figure 1d). This may be due to the smaller size of **P12+** as compared to **PS**, making it favorable for LMW heparin interaction.

We also compared the neutralizing potential of **P10+** towards low-molecular-weight (LMWH) and UFH heparins under physiological conditions (Tris-buffered saline, pH 7.40) via Anti-Xa assay. The results show **P10+** performed weaker than both **P12+** and **PS** towards UFH. Interestingly, **P10+** shows a better neutralization potential towards LMWH than **PS** but less than **P12+**, which can be attributed to its lower charge density (Figure S11). This confirms **P12+** as a better neutralizer for both UFH and LMWH.

We used confocal fluorescence microscopy (CFM) to visualize the real-time heparin-binding process. Incubation of **P12+** with Rhodamine-B-labelled heparin led to a distinguishable red fluorescence with a spherical shape (Figure 1e and Figure S12). Line graphs showed the time-dependent heparin scavenging effect of **P12+** towards the Rhodamine-B-labelled heparin (Figure 1f).

### 2.2 ∣ Methyl Orange as a Suitable Guest/Dye

**FIGURE 2 ∣:**
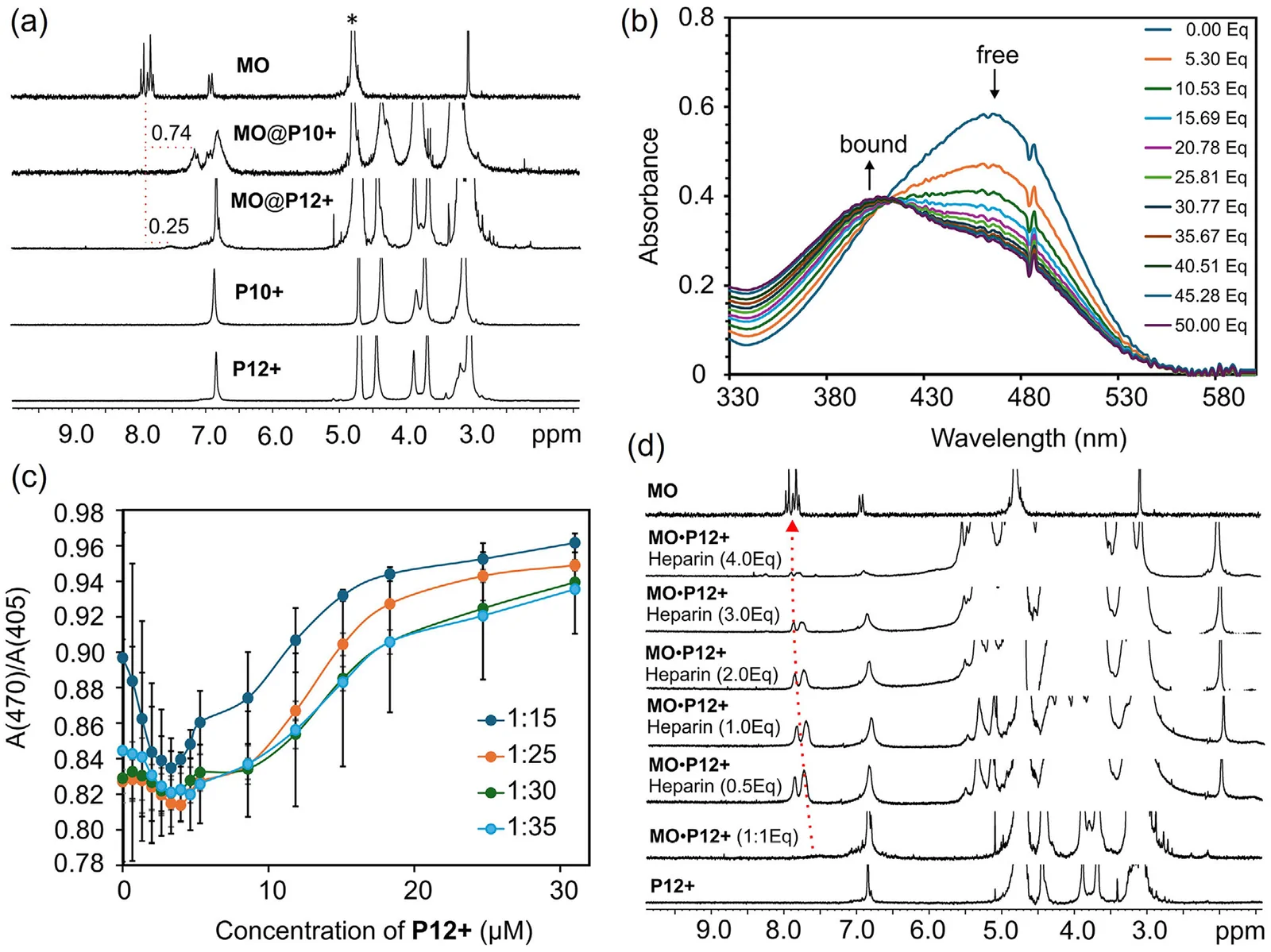
The complexation between the macrocycles (**P10+** and **P12+**) and the **MO** was studied by using NMR in D_2_O and UV–vis spectroscopy in physiologically relevant Tris-NaCl buffer (10 mM Tris, 150 mM NaCl, pH 7.4). (a) ^1^H NMR (D_2_O, 298 K) showing a clear shift of the aromatic signals of **MO**, more pronounced in host **P10+** than in **P12+**. (b) UV–vis titration showing a blue shift in the absorption spectra of **MO** when titrated with **P12+**. (c) By comparing the absorption maxima of free (470 nm) and bound (405 nm) **MO**, the host–guest concentration ratio with the most linearity was determined. The ratio of 1:25 was selected for heparin sensing measurements. (d) ^1^H NMR spectroscopy (D_2_O, 298 K) showing the release of **MO** from the cavity of **P12+** upon additions of heparin via monitoring aromatic signals of **MO**. The arrow shows shift changes indicating the release of the **MO** from P12+’s cavity upon titration of Heparin.

To test the potential of **P12+** as a sensor for heparin detection, we investigated the binding of the macrocycles **P12+** and **P10+** with **MO** as a potential sensor. Initially, the binding affinity of each macrocycle toward **MO** as a dye was evaluated using ^1^H NMR and ITC experiments. These methods have been used to study the interactions between **MO** and polycations [45], surfactants [46, 47], and macrocycles [3]. ^1^H NMR spectra of an equimolar mixture of **MO** and **P10+** revealed a 0.74 ppm shielding of the aromatic proton of **MO**. This significant shielding suggests in-cavity complexation of **MO** in the hydrophobic aromatic cavity of **P10+** via *π–π* interactions. Under identical conditions, the ^1^H NMR spectrum of an equimolar mixture of **MO** and **P12+** shows a modest shielding of 0.25 ppm (Figure 2a). To quantify this binding process, ITC experiments were done with results revealing a dissociation constant (*K*_d_) of 158 μM and 10.5 μM for the binding of **MO** by **P12+** and **P10+**, respectively (Table 1 and Figure S6). The binding of **MO** to **P10+** is a spontaneous reaction that is largely driven by enthalpy due to negative ΔH, in contrast to the binding of **MO** to **P12+**, which is only spontaneous at and beyond the experimental temperature and is mostly driven by entropy. This further confirms the favorability of **MO-P10+** interactions over **MO-P12+**. The higher binding affinity of **MO** by **P10+** is attributed to a better size complementarity that creates a good fit inside the narrow aromatic cavity of the macrocycle, as confirmed by both ^1^H NMR and ITC experiments. To further understand the binding of each macrocycle to **MO**, a UV–vis-based titration between the **MO** and the macrocycles was done. This method is also useful in analyzing **MO** interactions with polycations [3, 45, 48], cyclodextrins [49], palygorskite clays [50], and azo dyes [51]. The binding of the macrocycles to **MO** is characterized by a hypochromic–hypsochromic shift in the absorption spectra. A **P10+/MO** complex required up to two equivalents of the macrocycle to cause a significant hypochromic–hypsochromic shift in absorbance (Figure S13). However, **P12+/MO** complex required up to 50 equivalents to cause a significant hypochromic–hypsochromic shift in absorbance (Figure 2b, and Figures S14 and S15).

### 2.3 ∣ Heparin Sensing

**FIGURE 3 ∣:**
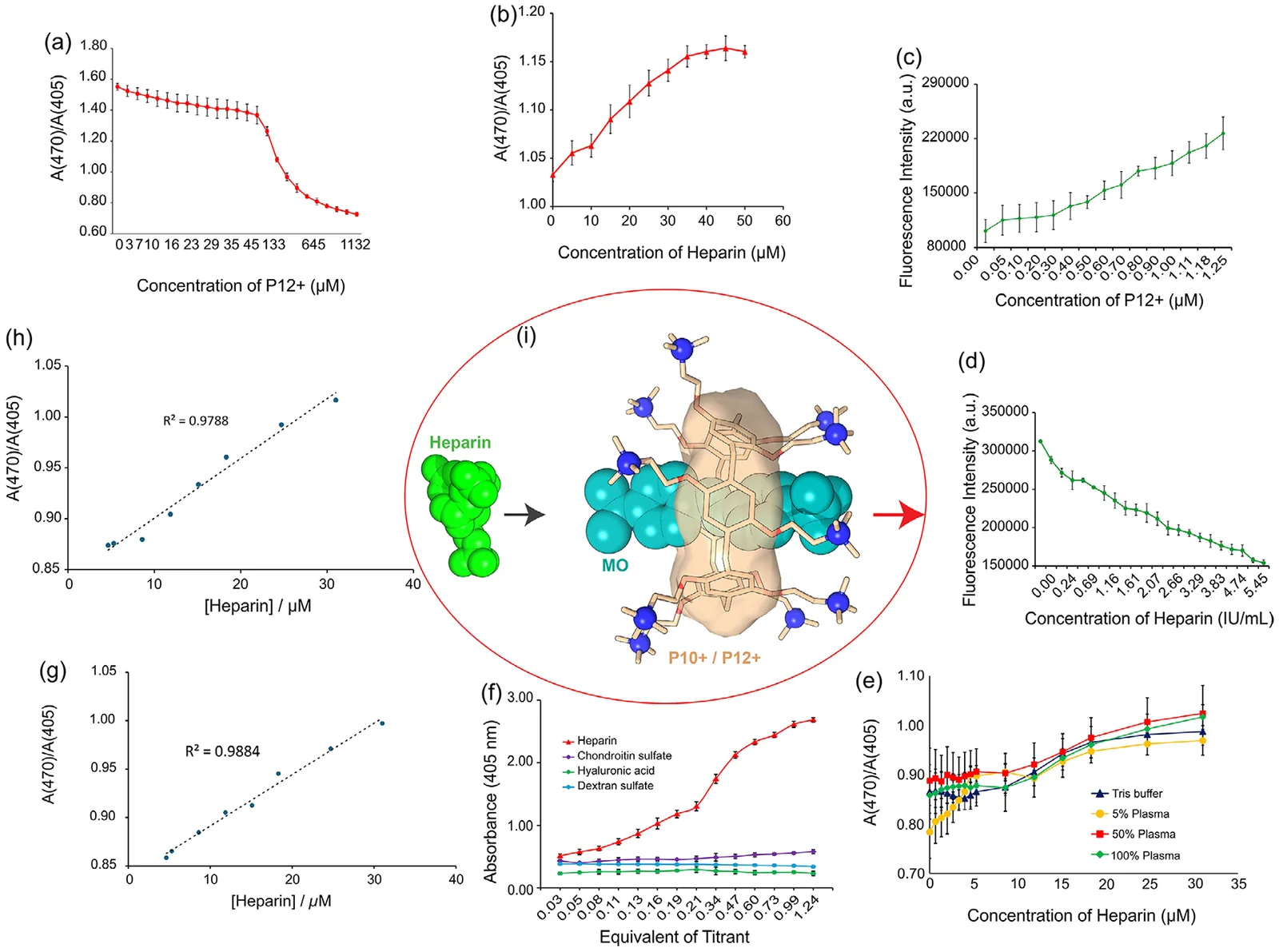
Heparin sensing ability of **P12+** was monitored in an IDA process using UV-vis and Fluorescence spectroscopy in physiologically relevant Tris-NaCl buffer (10 mM Tris, 150 mM NaCl, pH 7.4). By comparing the UV absorption maxima of free (470 nm) and bound (405 nm) **MO**, heparin levels were monitored relative to the release of **MO** from the complex. (a) Titration of **MO** into **P12+**, (b) titration of heparin into **P12+/MO** complex. By comparing the fluorescence absorption maxima of free (456 nm) and bound (450 nm) **MO**, heparin levels were monitored. (c) Titration of **MO** into **P12+**, (d) titration of heparin into **P12+/MO** complex. (e) Calibration curves for heparin detection in various media (Tris buffer and different concentrations of human plasma). (f) UV–vis showing **P12+**’s higher affinity for heparin as compared to other glycosaminoglycans (dextran sulfate, chondroitin sulfate, and hyaluronic acid). All experiments were done in triplicate. Data represent means ± standard error of the mean (S.E.M.). Calibration curve for UV–vis assay by comparing the absorption maxima of free (470 nm) and bound (405 nm) **MO**, the host–guest concentration: (g) in Tris Buffer, and (h) in 100% Plasma. (i) Schematic presentation of the host–guest binding between **MO** and **P12+** and the release of **MO** from the cavity in the presence of heparin. As heparin binds to **P12+**, it promotes the displacement of **MO** from the cavity of **P12+**. Color scheme: Heparin (green), Methyl Orange (Turquoise), P12+ (brown with the nitrogens in blue).

To further probe the **P12+** as a sensor for heparin detection, we investigated the kinetics of **MO** displacement from **P12+** and **P10+** in the presence of heparin. In a guest displacement assay monitored by ^1^H NMR titration, the heparin-scavenging ability of **P12+** was further investigated using **MO** as a reporter guest. This is observed by monitoring the ^1^H NMR signal of **MO** upon heparin titration into an equimolar mixture of **P12+** and **MO**, which led to the gradual displacement of **MO** from the internal cavity of **P12+** (Figure 2d). Displacing the **MO** from the internal cavity is a direct result of the higher affinity of heparin for **P12+**. This binding affinity is preferred over the encapsulation of **MO**, making **P12+** a potential chemical sensor for heparin. The guest displacement experiment positions **MO** as a potential dye in an IDA process for heparin sensing. This process has been employed in the detection and quantification of analytes such as white tea [52], amino acids [53], cellulose [54], cordycepin [55], among others, using various dyes. In an IDA process, a receptor–indicator complex is formed first, producing a measurable signal [56-58]. When a target analyte with higher affinity binds to the receptor, it displaces the indicator, causing a detectable change in the signal that can be used to identify or quantify the analyte. By comparing the absorption maxima of free (470 nm) and bound (405 nm) **MO**, the host–guest concentration ratio with the most linearity was determined. The ratio of 1:25 was selected for heparin sensing measurements, ensuring all the **MO** is bound at this ratio by **P12+** (Figure 2c and Figures S16-S19). A UV–vis spectroscopy-based IDA was employed, which produced approximately 80% release of **MO** for heparin detection (Figure 3a,b, and Figure S20). This is a marked increase compared to 40% release reported in a similar process with **P10+** [3]. The difference is largely due to a larger cavity size in **P12+**, which binds the **MO** more loosely than **P10+**. Another critical development with the use of **P12+** for heparin detection is its sensitivity in higher concentrations of plasma. Previous reports on **P10+** establish its sensitivity in a maximum of 5% plasma. However, we report calibration curves from UV–vis-based IDA assays in Tris EDTA buffer, 5%, 50%, and 100% human plasma, respectively (Figure 3e and Figures S20-S23). Calibration curves that cover the working linear range of this IDA process were plotted for heparin detection in Tris buffer and different plasma concentrations. (Figure 3g,h and Figures S27-S31). Spike and recovery experiments were done to ensure quantitative accuracy and eliminate any matrix effects during heparin detection in plasma. The recovery percentage in the different concentrations of plasma ranged from 84.8%–117% (Table S1). This indicates that the **P12+** sensor can specifically and accurately measure heparin despite any background noise in human plasma.

We probed the selectivity of **P12+** for heparin against other glycosaminoglycans in the body through a competitive displacement of **MO** from **MO-P12+** complex upon titration. Comparing absorbances at 405 nm for free **MO**, higher absorbances correlating with higher concentrations of **MO** were observed for the titration of heparin into a 1:25 **MO-P12+** complex, indicating a higher affinity for **P12+** than the affinity of **MO** for **P12+**. In contrast, titration of equimolar concentrations of dextran sulfate, chondroitin sulfate, and hyaluronic acid into a 1:25 **MO-P12+** complex produced very low absorbance values, indicating weaker interactions with **P12+** compared to heparin (Figure 3f and Figures S24-S26). This indicates that **P12+** has a higher affinity for heparin than for dextran sulfate, chondroitin sulfate, and hyaluronic acid. This behavior is attributed to the higher degree of sulfation and greater negative charge density of heparin, which also allows for a better conformational fit compared to chondroitin sulfate, which contains fewer sulfate groups and a lower charge density [59, 60]. Interestingly, the low affinity of **P12+** for dextran sulfate, despite the latter’s high sulfonate groups and charge density, demonstrates the macrocycle’s relative specificity toward heparin. Heparin is known to be relatively rigid, extended, and conformationally organized [61-64], while dextran sulfate is more flexible, highly branched, larger, and less conformationally ordered than heparin [65, 66]. This specificity could be a result of the conformation flexibility of dextran sulfate, which coils, and thus only a limited amount of the sulfonate groups are accessible to complex with **P12+**. Similar trends of interaction were observed in the interaction of heparin and dextran sulfate with mesoscopic cationic protein nanopores [67] and alkali ions [68]. Hyaluronic acid is non-sulfated and contains only one carboxyl group per disaccharide unit [69, 70], resulting in significantly lower negative charge density and, consequently, weaker affinity for **P12+**.

Fluorescence spectroscopy provides a more sensitive technique to detect therapeutic concentrations of heparin. To further analyze the sensitivity of **P12+**, fluorescence spectroscopy was employed for the IDA to analyze its ability to detect therapeutic doses of heparin (0.3–0.7 IU/ mL and 0.5–2.0 IU/ mL for UFH and LMW heparin, respectively). The detection range obtained falls within 0.11–5.75 IU/ mL, making the macrocycle suitable for the detection of both types of heparins under physiological conditions (Figure 3c,d and Figures S32-S33).

### 2.4 ∣ Toxicity

**FIGURE 4 ∣:**
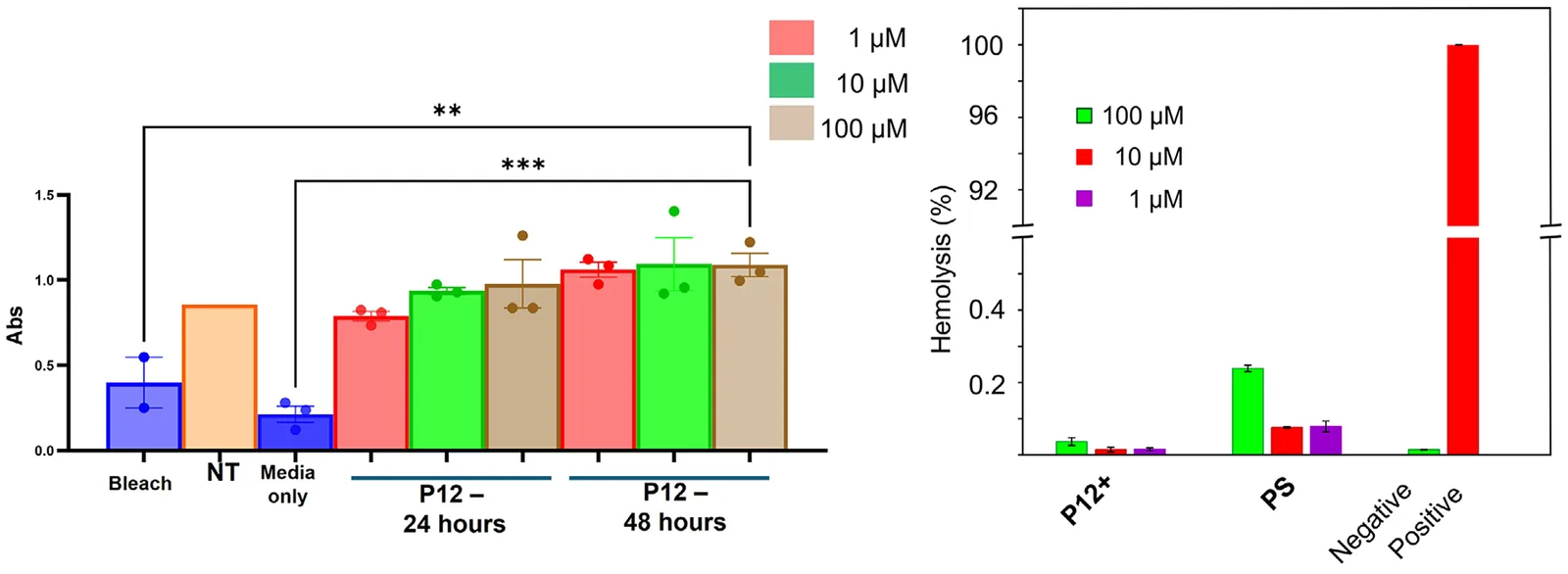
Biocompatibility assessments of **P12+** were determined by analyzing its cytotoxic and hemolytic effects. (a) MTT assays for 24 and 48 h using 1, 10, or 100 μM of the compound. (b) Percent hemolysis of RBC’s by **P12+** and **PS** in comparison to positive control (2% Triton) and negative control (PBS). Data are presented with means ± standard error of the mean (S.E.M.) (*n* = 3). *p < 0.05, **p < 0.01.

Successful heparin-scavenging requires that the scavenger be biocompatible. Hence, we evaluated the biocompatibility of **P12+** with hemolytic assay and 3-(4,5- dimethyl thiazol-2-yl)-2,5-diphenyl-tetrazolium bromide (MTT) dye cytotoxicity assay. HEK-293 cells were chosen for cytotoxicity evaluation because they are a well-established human-derived cell line that provides biologically relevant information regarding potential cellular responses to the material in a human context. Cell viability in response to treatment with compound **P12+** was assessed using an MTT assay at 24- and 48-h post-treatment (Figure 4a). Across both time points, treatment with increasing concentrations of **P12+** (1, 10, and 100 μM) generally did not result in significant cytotoxicity compared to untreated controls. Absorbance (Abs) values, which are proportional to the number of viable cells, remained relatively consistent around 1.0 for all concentrations tested, indicating minimal impact on cell viability. Viability was not affected at 48 h either. Control conditions, including media with no cells and bleach-treated wells, showed significantly reduced absorbance values, confirming the specificity of the MTT signal for viable cells. In contrast, media with cells and untreated control wells maintained high absorbance values, further validating assay performance. Together, these findings suggest **P12+** does not exhibit cytotoxicity in the tested concentration range over 24 and 48 h, supporting their potential compatibility in this in vitro model. Additionally, the toxicity was also tested through hemolytic assays, with results revealing no detectable toxicity to red blood cells up to 100 μM concentration of **P12+** and **PS** (Figure 4b).

### 2.5 ∣ Molecular Dynamics Simulations of the Indicator (MO) Displacement

**FIGURE 5 ∣:**
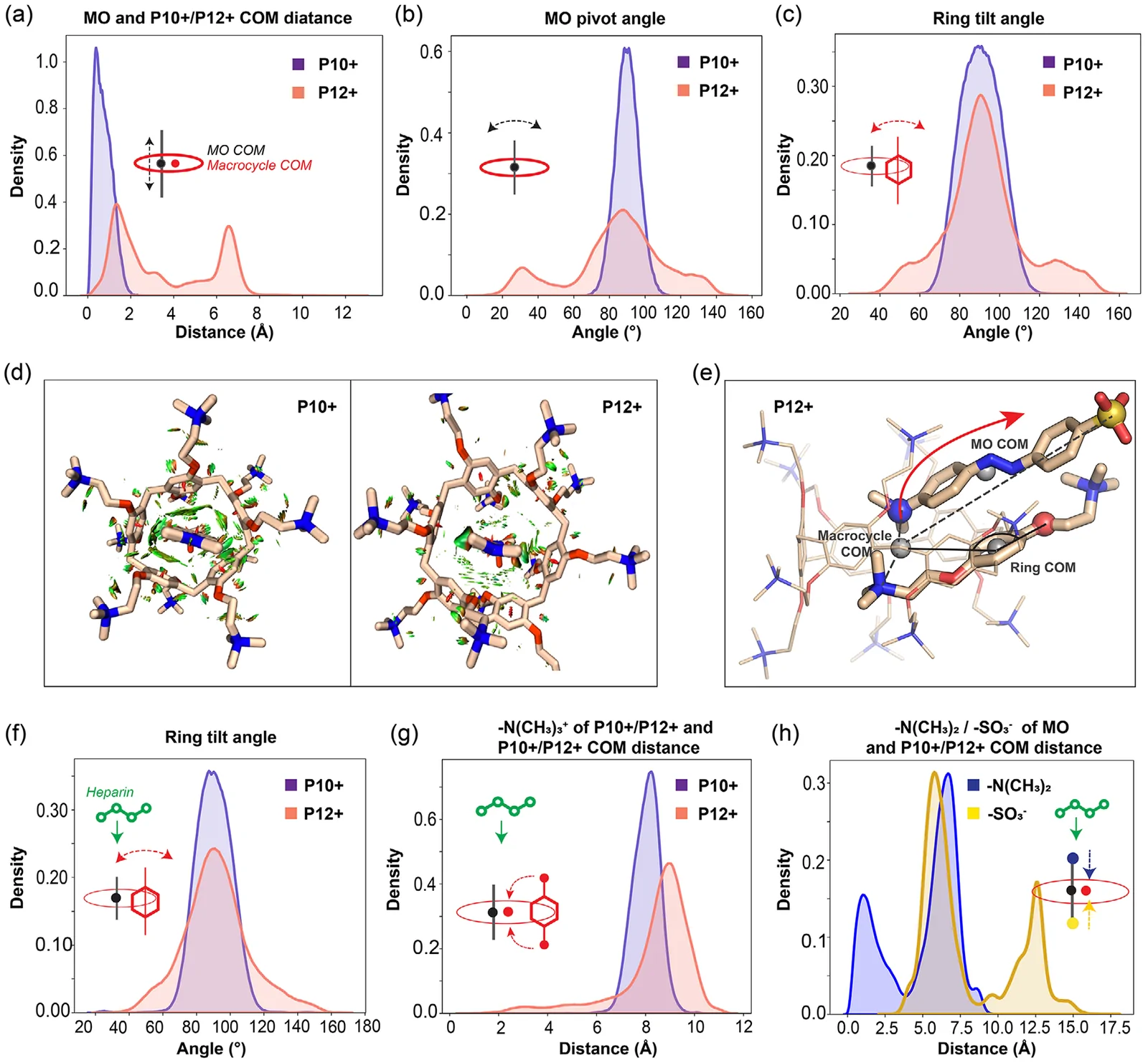
Trimethylammonium (-N(CH_3_)_3_^+^) groups of the P12+ facilitate MO to egress the macrocycle cavity. (a) Normalized density plot of the distance between the COM of the macrocycles and the COM of MO in the complexes. (b) Normalized density plot of the pivot angle of MO with respect to the plane of the macrocycles P10+ and P12+ to show the tilting oscillation of MO within the cavity. (c) Normalized density plots of the tilt angles of the pillararene units with respect to the plane of the macrocycles P10+ and P12+. (d) Non-bonded intermolecular interactions between MO and the macrocycles. Reduced density gradient (RDG) isosurfaces are colored according to $\text{sign}(\lambda_{2})\rho$: blue indicates attractive interactions, green corresponds to weak van der Waals interactions, and red represents steric repulsion. The intermolecular interactions are significantly weaker in P12+ than in P10+. (e) Snapshot of an intermediate conformation of MO escaping from P12+ captured by the steered molecular dynamics simulations. To escape from the cavity of the macrocycles, either the dimethylamino (–N(CH_3_)_2_) or the sulfonate (–SO_3_^−^) group of MO must pass through the center of the macrocycle (red arrow). Hence, the distance between the COM of the macrocycles and the dimethylamino (–N(CH_3_)_2_) and the sulfonate (–SO_3_^−^) groups are calculated. (f) Normalized density plots of the tilt angles of the flexible pillararene units with respect to the plane of macrocycles in the presence of a heparin octamer. (g) Normalized density plot of the distance between the COM of the macrocycles and the trimethylammonium (–N(CH_3_)_3_^+^) groups at the rims of the macrocycles P10+ and P12+. (h) Normalized density plot of the distance between the macrocycle COM and the dimethylamino (–N(CH_3_)_2_) and sulfonate (–SO_3_^−^) groups of MO.

To investigate the egress mechanism of **MO** from **P10+** and **P12+**, we performed MD simulations for both macrocycles in complex with **MO** and with **MO**–heparin. First, to evaluate **MO** stability within the **P10+:MO** and **P12+:MO** complexes, we calculated the center-of-mass (COM) distance between **MO** and the macrocycles. In the **P10+:MO** complex, **MO** remained largely stable within ~2 Å of the macrocycle COM. In contrast, in the **P12+:MO** complex **MO** resided either near ~2 Å or ~7 Å from the macrocycle COM, indicating greater mobility within the cavity of **P12+** (Figure 5a). To quantify the mobility of **MO**, we calculated the pivot angle of **MO** defined by the edge of the macrocycle ring, the COM of **MO**, and the sulfonate group of **MO**. The pivot angle distribution shows that **MO** is more constrained within **P10+** (70°–110°), whereas it samples a broader range in **P12+** (20°–140°) (Figure 5b). We further evaluated the flexibility of the pillararene units by calculating the tilt angle of the aromatic rings relative to the macrocycle plane. In **P10+**, the pillararene units remain approximately perpendicular (~90°) with a deviation of ±35°. In contrast, **P12+** shows larger fluctuations (~90° ± 65°), indicating greater ring flexibility compared to **P10+** (Figure 5c). The movement and flexibility of **MO** within **P10+** and **P12+**, as revealed by MD simulations, are consistent with the experimental thermodynamic behavior of the complexes, with tighter binding and reduced conformational freedom in **P10+** compared to **P12+** (Table 1 and Figure 5a,b). Subsequently, we quantified the interactions between the macrocycles and **MO. P10+** exhibits stronger interaction energy compared with **P12+** (Figure S34). The host–guest complexes are primarily stabilized by electrostatic interactions, which are stronger and more stable in **P10+** than in **P12+**. Semi-empirical tight-binding calculations further show stronger non-bonded interactions between **MO** and **P10+** than with **P12+**. Non-covalent interaction (NCI) analysis performed with Multiwfn produces colored reduced density gradient (RDG) isosurfaces between the interacting species, where the density of the isosurface represents the strength of the non-covalent interactions. In the **P12+:MO** complex, the thinner RDG isosurface indicates weaker intermolecular interactions than those observed for the **P10+:MO** complex (Figure 5d). The weaker interactions in **P12+**, therefore, could allow greater **MO** flexibility and facilitate its egress compared with **P10+**.

To further probe the egress mechanism, we performed SMD simulations in which a heparin octamer (4 × IdoA(2S)–GlcNS(6S)) was placed 25 Å from the macrocycle COM and pulled toward the macrocycle center (Figure S25). This setup allows characterization of the **MO** escape mechanism as heparin approaches the macrocycle. To identify which functional group, either the dimethylamino (─N(CH_3_)_2_) or the sulfonate (─SO_3_^−^) of **MO** exits the macrocycle, heparin was positioned on both sides of **MO**. The distance restraint was applied to the macrocycle COM, allowing **P10+/P12+** to rotate and to engage favorably with the heparin octamer (Figure S35), without restricting orientation or direction. Irrespective of the direction from which heparin approaches **P10+/P12+** macrocycles, **MO** escaped only when its sulfonate (─SO_3_^−^) group moved out of the macrocycle cavity. Since the restraint on the macrocycle COM does not restrict its orientation, the macrocycle rotates and engages with heparin either through the trimethylammonium groups on the dimethylamino (–N(CH_3_)_2_) side of **MO**, or through the macrocycle aromatic rings. In most of the **P12+** simulations (17 of 24 SMD simulations), **MO** displacement occurred, whereas in the **P10+** simulations, only 4 simulations showed **MO** displacement, irrespective of the force used in SMD simulation (Movie S1). During the SMD simulations, the trimethylammonium (–N(CH_3_)_3_^+^) groups at the rims of **P12+** were observed to move toward the macrocycle center. To elucidate this behavior, we analyzed two parameters: (i) the tilt angles of the macrocycle rings to evaluate the influence of heparin on the pillararene units; and (ii) the distance between the macrocycle COM and the trimethylammonium (–N(CH_3_)_3_^+^) groups at the rims. In addition, the distance between the macrocycle and the dimethylamino (–N(CH_3_)_2_) and sulfonate (–SO_3_^−^) groups of **MO** is calculated to determine the direction of **MO** escape (Figure 5e). Because the dimethylamino and sulfonate groups lie on opposite sides of the macrocycle, either one of these groups must pass through the macrocycle COM during **MO** release (Figure 5e).

The tilt-angle distributions show that the macrocycles exhibit greater ring flexibility in the presence of heparin. In unbiased MD simulations, the **P10+** pillararene rings tilt within a range of 56.0–131.6°, whereas **P12+** spans 26.8–161.4°. In the presence of heparin, these ranges expand by 63° and 17.0°, to 16.4–154.8° for **P10+** and 19.6–170.8° for **P12+**, respectively (Figure 5c,f). The increased flexibility of the pillararene rings in the presence of heparin facilitates movement of the trimethylammonium groups at the rims to move closer toward the center of the macrocycles. Analysis of the trimethylammonium (–N(CH_3_)_3_^+^) and macrocycle COM distance shows that in **P12+** these groups can move as close as ~0 Å to the macrocycle center, whereas in **P10+** they remain constrained at distances ~6 Å (Figure 5g). To evaluate the effect of this motion, we analyzed the distances between the macrocycles and the dimethylamino and sulfonate groups of **MO**. The results show that only the dimethylamino (–N(CH_3_)_2_) group approaches the macrocycle COM, whereas the sulfonate group does not move closer than ~3.5 Å (Figure 5h and Figure S36). This indicates that the sulfonate group exits the macrocycle first, while the dimethylamino group is pushed by the cationic trimethylammonium (–N(CH_3_)_3_^+^) groups of the macrocycle rims. Mechanistically, the sulfate groups of heparin interact with the trimethylammonium groups at the pillararene rims, primarily positioned near the dimethylamino (–N(CH_3_)_2_) group of **MO**, which in turn pushes the dimethylamino (–N(CH_3_)_2_) group of **MO**, promoting its escape from the macrocycle cavity (Figure S36). This process is primarily favored in **P12+** due to its larger cavity and weaker host–guest interactions than those of **P10+**.

## 3 ∣ Conclusion

We have proven that **P12+** forms stable complexes with heparin and exhibits strong neutralization ability towards both UFH and low-molecular-weight heparins in Tris buffer and in human plasma. **P12+** shows higher affinity towards heparin when compared to other glycosaminoglycans in the body (chondroitin sulfate and hyaluronic acid). This is attributed to fewer anionic groups and, as such, weaker interactions with **P12+**. Interestingly, **P12+** shows less affinity towards dextran sulfate, a highly polysulfonated sugar, which is not a glycosaminoglycan found naturally in the human body. We speculate that conformation flexibility of dextran sulfate could result in less availability of the sulfonate groups for interaction with **P12+**. We demonstrated that the **P12+** macrocycle affords detection of therapeutic concentrations of heparin in human plasma, confirmed through UV–vis and fluorescence spectroscopy-based IDA process. The heparin detection ability of **P12+** was found to be more pronounced than that of the previously reported **P10+**. Cytotoxicity measurements through MTT dye and hemolytic assays suggest **P12+** does not exhibit cytotoxicity in the tested concentration range over 24 and 48 h, supporting their potential compatibility in this in vitro model. Computational results complement and support the experimental findings and successfully rationalize the **MO** egress mechanism. Simulations demonstrate that the efficiency of **P12+** is associated with weaker interactions and increased flexibility of **MO** within **P12+**, while the cationic groups at both rims of **P12+** facilitate **MO** egress. Overall, the computational study provided a molecular–level understanding of the efficient release of **MO** from the **P12+** cavity, making it a more suitable agent for heparin sensing.

## 4 ∣ Experimental Section

### 4.1 ∣ Materials

**PS**, UFH heparin, low molecular weight heparin, methyl orange, dextran sulfate, chondroitin sulfate, hyaluronic acid, and other synthetic reagents were purchased from Sigma-Aldrich. All the solvents used were purchased from Thermo Fisher Scientific and Sigma Aldrich, USA.

### 4.2 ∣ Ethics

All studies using human subjects were approved by the Oakland University Institutional Review Board based on the Declaration of Helsinki (IRB-FY2021-390). The blood samples were obtained with prior informed consent and approval from the institutional ethics committee.

### 4.3 ∣ Synthesis

Receptors **P12+** and **P10+** were synthesized according to reported procedures [71]. Briefly: The precursor 1,4-bis (2-bromoethoxy)benzene was synthesized from hydroquinone in the presence of carbon tetrabromide, 1,2-dibromoethane, triphenylphosphine in dry acetonitrile. 1,4-bis(2-bromoe thoxy)pillar[5]arene and 1,4-bis(2-bromoethoxy)pillar[6]arene were synthesized from the Lewis acid-catalyzed cyclo condensation reaction of 1,4-bis(2-hydroxyethoxy)benzene and paraformaldehyde. 1,4-bis(2-bromoethoxy)pillar[5]arene was obtained in chloroform while 1,4-bis(2-bromoethoxy)pillar[6]arene was obtained in cyclohexylchloride. **P10+** and **P12+** were obtained in the presence of trimethylamine in ethanol.

### 4.4 ∣ NMR Spectroscopy

^1^H NMR was employed to determine the differences in affinity of **MO** towards **P12+** and **P10+**, respectively, to understand the kinetics of the IDA with either of the macrocycles. The affinity was assessed through a host–guest interaction between **MO** and the macrocycles. ^1^H NMR of pure compounds was compared to a 1:1 mixture of the compounds. For measurements of pure compounds, 5 mM of each of **P12+, P10+**, and **MO** was prepared in deuterium oxide (D_2_O). A 250 μL proportion was put in an NMR tube and diluted to 500 μL with deuterium oxide. For host–guest mixtures, 250 μL each of **P12+** and **MO** was measured into an NMR tube. This was repeated for **P10+** and **MO**. ^1^H NMR measurements were performed using an Avance 400 MHz Bruker Spectrometer.

To study the displacement of **MO** by heparin using ^1^H NMR, aliquots of stock heparin solution in deuterium oxide (D_2_O) were titrated into a 1:1 equivalent mixture of **P12+** and **MO** in D_2_O to achieve 0.5, 1.0, 2.0, 3.0, and 4.0 equivalents of heparin. ^1^H NMR measurements were done on a 400 MHz Bruker Spectrometer.

### 4.5 ∣ ITC

The ITC experiment was carried out by filling the sample cell with either **P12+** or **P10+** (1 mM), filling the syringe with **MO** (10 mM) or heparin, and titrating via computer-automated injector at 310 K. Blank titrations into plain Tris buffer (10 mM/ 150 mM) were also performed and subtracted from the corresponding titration to remove any effect from the heats of dilution from the titrant. Isotherms and thermodynamic parameters from an independent fitting model (K_d_, ΔH, and ΔS) were obtained using the NaNoAnalyze software. Gibbs’ free energy Δ*G* was subsequently calculated at 310 K and recorded. Measurements were done using the Nano ITC instrument by TA Instruments.

### 4.6 ∣ Dynamic Light Scattering (DLS)

To assess the size and count rates of binder-heparin complexes, dynamic light scattering experiments were employed in TRIS/NaCl buffer. Different equivalents of **P12+** were titrated into 1 mL heparin solution up to 6 mass equivalents. The derived count rate and hydrodynamic diameters were derived from triplicate experiments. This was repeated for **P10+** and **PS**, respectively. Measurements were done using the Malvern zetasizer instrument.

### 4.7 ∣ Anti-Xa

Neutralization potential of **P12+, P10+**, and protamine sulfate in plasma was studied by using a commercial two-stage kit, Biophen Heparin Anti-Xa. Samples were prepared by adding heparin (0.1 mg/ml, 10 mM Tris-150 mM NaCl) to human plasma, followed by neutralizers in different concentrations up to 3 molar equivalents. Samples were diluted, and the kit’s reagents were reconstructed according to the manufacturer’s instructions. Final concentration of heparin was 0.069 units/ml, and the mass ratio of host-to-heparin ranged between 0.5 and 3. To run the calorimetric assay, 40 μL sample solution and 40 μL antithrombin were added to wells of a 96-well microplate and incubated for 2 min. This was followed by the addition of factor Xa (40 μL), 2 min incubation, addition of the chromogenic substrate (40 μL), and a further 2 min incubation. Reaction was stopped by introducing 80 μL of 2% citric acid. Absorbance at 405 nm was measured immediately using the Biotek Cytation 3 microplate reader. Anticoagulant activity is inversely proportional to the absorption intensity [3, 25, 72, 73]. The percentage of neutralization was determined with the help of a calibration curve of heparin performed according to the manufacturer’s instructions. Assay was performed three times, and averages with standard deviations are presented.

### 4.8 ∣ CFM

Fluorescence data of heparin scavenging by **P12+** were acquired using a Nikon Eclipse *Ti* confocal laser scanning microscope equipped with solid-state lasers at 561 nm for rhodamine detection. The measurements were performed with a ×10/0.40 numerical aperture (NA) (1.55 × 1.55 mm^2^ field of view, 7 μm slice thickness) objective at a resolution of 512 × 512 pixels. The photon counting mode of the hybrid detector was used. To measure heparin uptake by **P12+**, 3 μL of 0.1 mg/mL **P12+** solution and 1 μL of Rhodamine B-labeled heparin (0.1 mg/mL) followed by the addition of Tris-NaCl buffer to a final volume of 10 μL (pH 7.4, Tris (10 mM), NaCl (150 mM)). The suspension was incubated at room temperature and then imaged over time (0, 30 and 60 min) on a glass slide using a confocal laser scanning microscope.

### 4.9 ∣ UV–Vis Spectroscopy

UV–vis spectroscopy was used to first determine the appropriate receptor-methyl orange concentration ratio for heparin detection. Titrations were performed with **MO-P12+** ratios of 1:15, 1:25, 1:30, and 1:35, respectively. Ratios of absorbances of free (470 nm) and bound (405 nm) methyl orange were plotted to find the most linear graph for heparin detection. Subsequently, UV–vis spectroscopy was employed in an IDA process for the detection of heparin. A 1:25 mixture of methyl orange and **P12+** was titrated with aliquots of heparin. This was compared to a titration of **P12+** into **MO** in the absence of heparin. A ratiometric plot of free (470 nm) and bound (405 nm) heparin was plotted to determine the amounts of heparin detected and the overall sensitivity of the IDA process for heparin. Experiments were performed in triplicate in Tris-NaCl buffer. Calibration curves were determined for 5, 50, and 100%v/v human plasma. UV–vis absorbance measurements were done using the Cary series UV–vis spectrophotometer by Agilent Technologies. Calibration curves for heparin detection using this assay were plotted for the linear range of the assay method (5–30 μM of heparin). To prove the accuracy of heparin detection with **P12+** in plasma and eliminate any matrix effects, a spike and recovery experiment was conducted in 5%, 50% and 100% plasma against 100% Tris/NaCl buffer samples. A stock concentration of heparin was prepared in 1 mL each of 5%, 50% and 100% human plasma as well as 100% Tris/NaCl buffer. Aliquots of each were titrated into a 1:25 molar equivalent mixture of **MO:P12+** to achieve a 10 μM (low concentration), 20 μM (medium concentration), and 30 μM (high concentration) of heparin selected from the linear range of the assay method. Similar concentrations of heparin were prepared in Tris/NaCl buffer and titrated into a 1:25 mixture of **MO:P12+**. Actual concentrations of heparin in the different plasma concentrations were deduced from the absorbance calibration curves and calculated as a percentage of concentrations in Tris/NaCl buffer. Experiments were done in triplicate.

### 4.10 ∣ Fluorescence Spectroscopy

Fluorescence spectroscopy affords a more sensitive IDA process to detect clinical amounts of heparin in physiological solutions. A 1:25 mixture of methyl orange and **P12+** was titrated with aliquots of heparin. This was compared to a titration of P**12+** into methyl orange in the absence of heparin. Absorbances of free (456 nm) and bound (450 nm) methyl orange were plotted. Experiments were done in triplicate. Fluorescence emission spectra were obtained using the Nanolog modular spectrofluorometer by HORIBA.

### 4.11 ∣ Hemolysis Assay

Human red blood cells from donors were used for this assay. Whole blood was centrifuged at 500 × g to remove platelets. The resulting supernatant was subsequently centrifuged at 16 000 × g for 10 min at 4°C, and the clarified plasma was collected for subsequent experiments. The cells were washed with 1× PBS three times and redispersed to the initial volume in 1× PBS. The cells were diluted 25× and split into 96-well culture plates (100 μL/well). The concentrated sample solutions (100 μL) in 1×PBS were added to each well, resulting in the desired final compound concentrations (1, 10, and 100 μM). One hundred microliters of 2% Triton X-100 in 1× PBS and 100 μL of 1× PBS were added as positive and negative controls, respectively. After incubation at 37° C for 1 h, the plates were centrifuged for 5 min at 500 × g to pellet intact erythrocytes, and 100 μL of the supernatant from each well was cautiously transferred into a clear 96-well plate. The resulting hemoglobin in the supernatant was measured at 540 nm with a microplate reader (Cytation 3, Biotek). The measurements were performed using triplicate samples. The percentage of hemolysis was calculated as follows:

$$\%\text{Hemolysis}=[(A_{\text{sample}}-A_{\text{negativecontrol}})\;\;\;∕(A_{\text{positivecontrol}}-A_{\text{negativecontrol}})]\times 100$$

### 4.12 ∣ Cytotoxicity

Human embryonic kidney cells (HEK-293, ATCC CRL-1573) were cultured in Dulbecco’s Modified Eagle Medium (DMEM) supplemented with 10% fetal bovine serum (FBS) and 1% penicillin-streptomycin. Cells were maintained at 37° C in a humidified incubator with 5% CO_2_ and passaged at approximately 70%–80% confluency using 0.25% ethylenediaminetetraacetic acid (EDTA)-trypsin. Cells were seeded into 96-well plates at a density of 2 × 10^4^ cells per well (100 μL of a 2 × 10^5^ cells/mL suspension) and allowed to adhere overnight. Compound **P12+** was prepared at final concentrations of 1, 10, and 100 μM and added to the wells in triplicate for each concentration. Treatment groups were incubated for either 24 or 48 h. Media were refreshed at the 24-h mark for the 48-h treatment groups to maintain cell viability. At the end of each treatment period, 10 μL of MTT reagent (prepared by dissolving 15 mg of MTT powder in 3 mL of sterile double-distilled water) was added to each well. Plates were incubated for an additional 4 h at 37° C to allow for formazan crystal formation. Following incubation, 100 μL of STOP solution (prepared from 40 mL 2-propanol, 5 mL 1N HCl, and 5 mL Triton X-100) was added to each well to solubilize the formazan crystals. Plates were then incubated for 6 h or overnight at room temperature. Absorbance was measured using a TECAN plate reader at 570 nm, with background correction at 690 nm. Data were analyzed to assess percent viability relative to untreated controls and to generate dose–response curves. Measurements were carried out using triplicate samples.

### 4.13 ∣ Molecular Dynamics Simulation

The 3D structures of pillar[5]arene (**P10+**), pillar[6]arene (**P12+**), and Methyl orange (MO) were drawn and optimized using MarvinSketch and Avogadro, respectively [74, 75]. **MO** was placed and positioned at the center of **P10+** and **P12+**. The systems were solvated in an orthorhombic box with added TIP3P water molecules using a padding size of 12 Å. All simulations were conducted using the AMBER20 [76], where the Sander.MPI module was used for minimization, and the pmemd.cuda module was used for equilibration and production simulations. The force field parameters for **P10+, P12+**, and **MO** were generated using Antechamber with the GAFF2 force field [77].

Each system was energy minimized in two steps: 20 000 steps of restrained minimization followed by 20 000 steps of unrestrained minimization. In each step, the first 5000 steps were performed using the steepest descent method, and the remaining steps used the conjugate gradient method. After minimization, the systems were then heated from 0–100 K over 3 ns, after which the temperature was increased to 303 K over 6 ns, followed by 10 ns of NPT equilibration. The production run involved 500 ns of MD simulation under NPT conditions with a 2 fs time step. The temperature was maintained at 303.15 K using a Langevin thermostat, the pressure was maintained at 1 atm using a Berendsen barostat, long-range interactions treated using particle mesh Ewald (PME) method with a10 Å cutoff for nonbonded interactions, and bonds involving hydrogen constrained using the SHAKE algorithm [78, 79]. Each simulation was performed in three replicates.

### 4.14 ∣ SMD With Heparin

To assess the mechanism of **MO** egress from **P10+** and **P12+**, we performed SMD in the presence of a heparin octamer. The initial structures were minimized for 5000 steps using the steepest descent method, followed by 20 000 steps using the conjugate gradient method. The systems were then heated from 0–100 K over 3 ns, after which the temperature was increased to 303 K over 6 ns. The systems were equilibrated for 10 ns using semiisotropic NPT condition. The heating and the equilibration are performed with positional restraints applied to the heparin placed 25 Å from the center of mass of **P10+/P12+** to maintain the heparin above the macrocycle. After equilibration, SMD was performed with a pulling rate of 1 Å/ns using a range of force constants (1, 2, 5, and 10 kcal/mol/Å and three replicates for each force constant) to move the heparin toward the core of **P10+** and **P12+**. Simulations were conducted by placing the heparin on both sides of methyl orange (dimethylamino (–N(CH_3_)_2_) and sulfonate (–SO_3_^−^) group sides) (24 independent SMD simulations in total). Because the restraints were applied to the center of mass (COM) of **P10+/P12+**, the molecules retained rotational freedom (Figure S22). Trajectories were recorded every 10 ps during the SMD simulations. All simulations were conducted using the AMBER20 software package with a time step of 2 fs. Long-range electrostatics treated using PME with a 10 Å cutoff and bonds involving hydrogen were constrained using SHAKE algorithm. The Sander.MPI module was used for minimization, and the pmemd.cuda module was used for equilibration and production simulations [76].

### 4.15 ∣ Trajectory Analysis

The analysis included all production trajectories (1.5 μs) from the unbiased simulations. Additionally, all SMD trajectories (24 independent trajectories) were considered for the analysis of the SMD simulations. Distance and tilt-angle analyses were performed using cpptraj from AmberTools23 [80]. The NCI analysis was conducted on the optimized structures of the P10+:MO and P12+:MO complexes, obtained at the HF/6-31G* level of theory using Gaussian 16 [81]. NCI plots were generated with Multiwfn [82, 83], and visualized using Visual Molecular Dynamics package (VMD 1.9.3) [84], with isovalues of 0.6 for both systems. The Matplotlib Python library and PyMOL (2.3.2) were used for plotting and structural visualization [85, 86].

## Supplementary Material

Movie1

SI

Additional supporting information can be found online in the Supporting Information section.

**Supporting File 1**: smll74588-sup-0001-SuppMat.pdf.

**Supporting File 2**: smll74588-sup-0002-MovieS1.pptx.

## Figures and Tables

**TABLE 1 ∣:** Thermodynamic parameters associated with the binding of heparin and **MO** by the macrocycles **P10+** and **P12+** in Tris/NaCl buffer at 310 K.

Complex	*K*_d_ (× 10^−6^) M	ΔH kcal/mol	TΔS kcal/mol	ΔG kcal/mol
**Heparin@P10+**	13.72 ± 3.70	3.01 ± 0.44	10.42	−7.41
**Heparin@P12+**	4.02 ± 0.11	4.94 ± 0.17	12.71	−7.77
**MO@P10 +**	10.50 ± 1.58	−6.94 ± 0.05	−0.14	−6.80
**MO@P12 +**	158.00 ± 38.61	3.44 ± 0.51	10.34	−6.90

## Data Availability

The data that support the findings of this study are available from the corresponding author upon request.
