# Supplementary material for: Engineering Host–Guest Interactions for Next-Generation Heparin Biosensors
Source: Small. Author manuscript; Available in PMC 2026 Aug 25. (PMC13501547; doi:10.1002/smll.74588)
Supplement: Supplementary file 1 [file NIHMS2201956-supplement-SI.pdf]

Supporting Information

Engineering Host–Guest Interactions for Next-Generation Heparin Biosensors

Frank Boateng Osei,[a] Charles Mariasoosai,[b] Zackary Christodolu,[a] Chloe Caringi,[c] Sahar Heidari,[b] Kwaku Twum,[a,d] Sithara Nanjappa,[c] Evan Trivedi,[a] Valance Washington,[c] Hedieh Torabifard,[b] and Ngong Kodiah Beyeh*[a]*

F. B. Osei, Z. Christodolu, K. Twum, E. Trivedi, N. K. Beyeh
Department of Chemistry, Oakland University, 146 Library Drive, Rochester, MI 48309-4479, USA
E-mail: beyeh@oakland.edu
C. Mariasoosai, S. Heidari, H. Torabifard
Department of Chemistry and Biochemistry, The University of Texas at Dallas, 800 West Campbell Road, Richardson, Texas 75080-3021, USA
C. Caringi, S. Nanjappa
E-mail: Hedieh.Torabifard@utdallas.edu
Department of Biological Sciences, Oakland University, 118 Library Drive, Rochester, MI 48309-4452, USA
K. Twum
Department of Pathology and Laboratory Medicine, Boston University School of Medicine, Boston, MA, USA

Contents

I. General Information 2
II. Synthesis 2
III. Isothermal Titration Calorimetry (ITC) 6
IV. Dynamic Light Scattering (DLS) 7
V. Anti-Xa 10
VI. Confocal Fluorescence Microscopy (CFM) 12
VII. UV-Vis and Fluorescence Spectroscopy 13
VIII. Molecular Simulations 34
VIII. References 35

I. General Information

Receptors **P12+** and **P10+** were synthesized according to reported procedures[1]. Protamine sulfate, unfractionated heparin, and low molecular weight heparin were purchased from Sigma-Aldrich. All the solvents used were purchased from Thermo Fisher Scientific and Sigma Aldrich, USA. Anti-Xa assay and dynamic light scattering experiments were carried out in plasma and 10mM TRIS buffer, respectively using TAKE 3 BioTek microplate reader and Malvern Zetasizer, respectively. ^{1}H NMR was carried out on 400MHz Bruker Spectrometer. ITC measurements were carried out using NanoITC instrument from TA Instruments. UV-Vis and fluorescence spectroscopy measurements were carried out on Cary Series UV-Vis spectrophotometer by Agilent Technologies and Nanolog®modular spectrofluorometer by HORIBA, respectively. Confocal fluorescence microscopy measurements were carried out using Nikon Eclipse T*i* confocal fluorescence microscope.

II. Synthesis

Compound **1** was purchased from Sigma Aldrich. Compounds **2-4**, **P10+**, and **P12+** were synthesized according to reported procedures.[1]

Carbon tetrabromide (39.8 g, 120.0 mmol) was slowly added in small portions to a solution of 1,4-bis(2-hydroxyethoxy)benzene (10.0 g, 50.4 mmol) and triphenylphosphine (31.5 g, 120.0 mmol) in 300 mL of dry acetonitrile at 0 °C with stirring. The reaction mixture was allowed to warm to room temperature, and the resulting clear solution was stirred for another 4 h under N2. Then 200 mL of cold water was added to the reaction, upon which product **1** precipitated as a white solid. The product was collected by vacuum filtration, thoroughly washed with methanol/water 60:40, and then recrystallized from methanol, filtered, dried to give compound **2** as white flakelike crystals, yield 13.8 g (85%). 300 mL of the solvent (chloroform for pillar[5]arene and cyclohexylchloride for pillar[6]arene) was added to 6 g of compound **2** while stirring. Paraformaldehyde (0.96 g) and boron trifluoride etherate (6 mL) were added to the solution and stirred for 2 hours at 25 °C. Water (150 mL) was added and stirred for a few minutes. The organic layer was removed and dried with sodium sulfate. The solvent was evaporated to yield the pillar[n]arene, **5** and **6**, respectively. Compound **3** or **4** was dissolved in methanol (100 mL) and trimethylamine (40 mL) and refluxed for 24 hours at 80 ^{o}C. The formed precipitate was rinsed with dichloromethane (50 mL), filtered, and dried to yield **P10+** and **P12+**.

P12+: m.p. > 300 ^{o}C; ESI-TOF-HRMS: $(C_{102}H_{180}O_{12}N_{12}Br_9)^{+3}$ [**P12+**-3Br]$^{+3}$, *m/z* Found 825.2178; calc. 825.2159. ^{1}H NMR (400 MHz, 298 K in D_2O) δ (ppm): 3.05 (br, 108H, $N(CH_3)_3$), 3.68 (br, 24H, NCH_2), 3.88 (br, 12H, Ar-CH_2), 4.44 (br, 24H, OCH_2), 6.84 (br, 12H, ArH); ^{13}C NMR: (100 MHz, 298 K in D_2O) δ = 30.08, 54.00, 63.34, 65.06, 116.18, 129.04, 149.76.

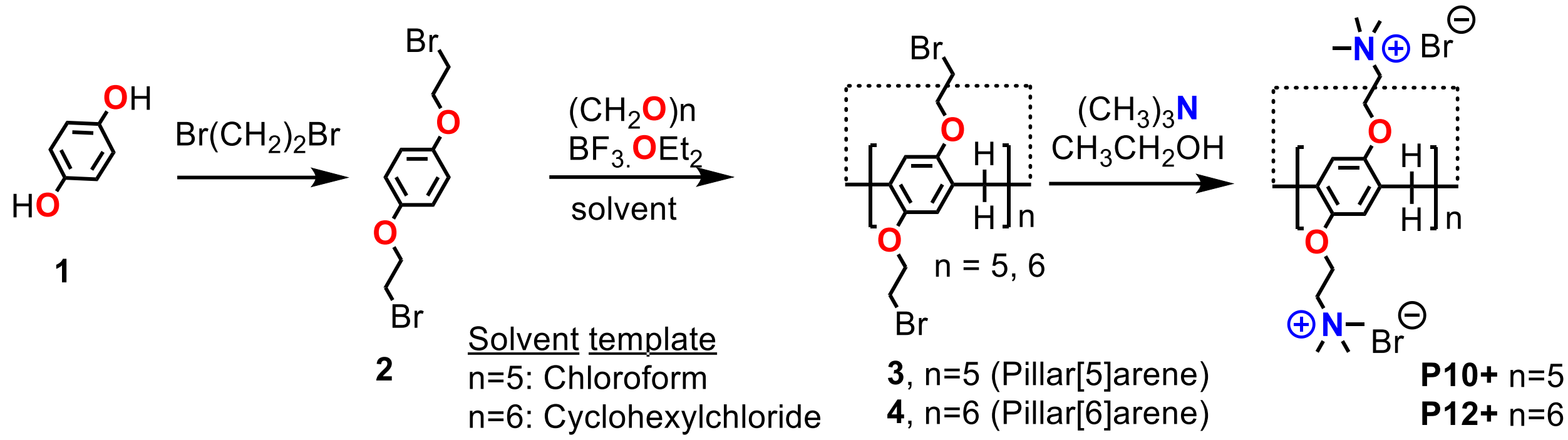

Figure S1. Synthesis of **P10+** and **P12+** from precursors.

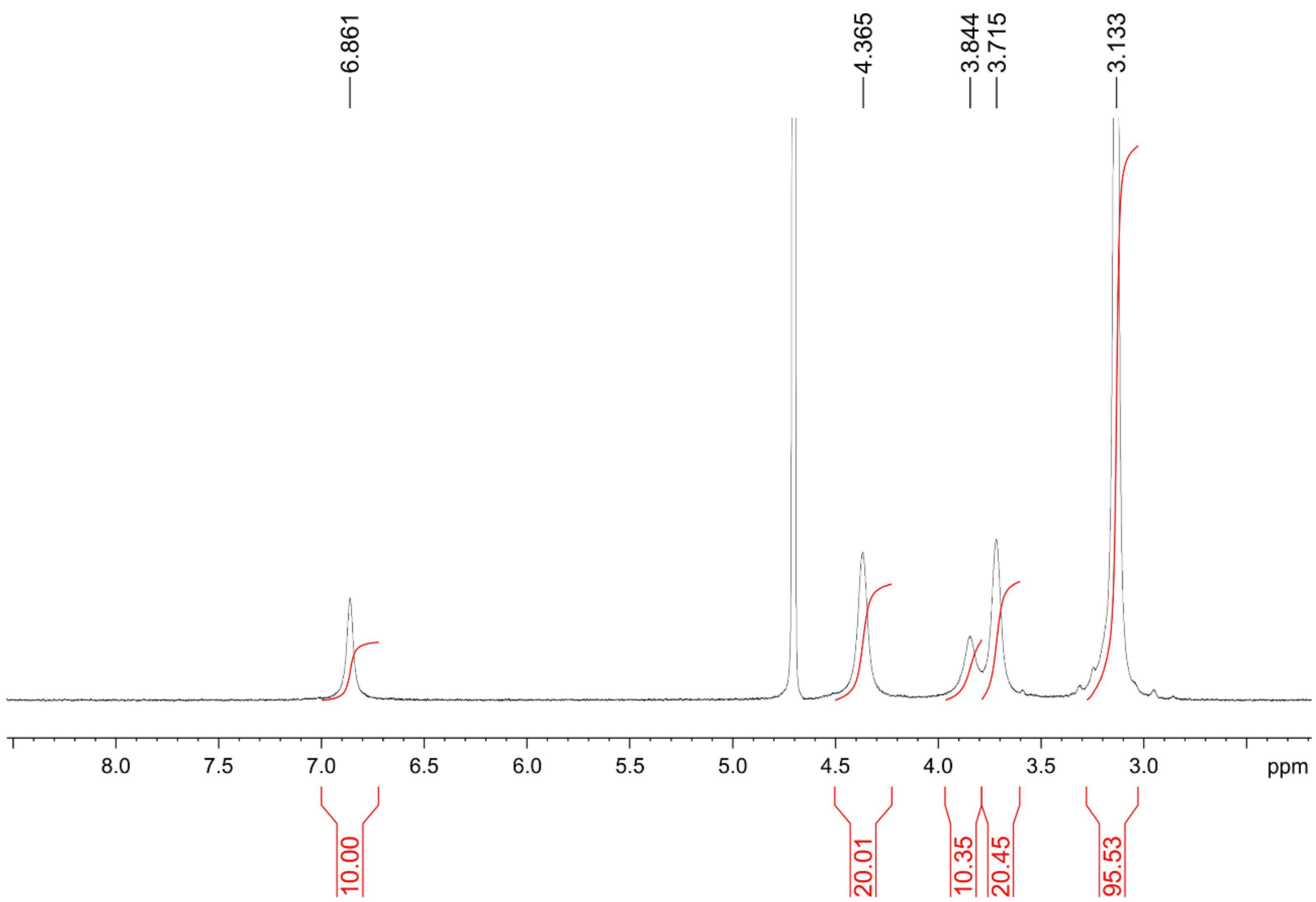

Figure S2. ^{1}H NMR of **P10+** in D_2O at 298 K.

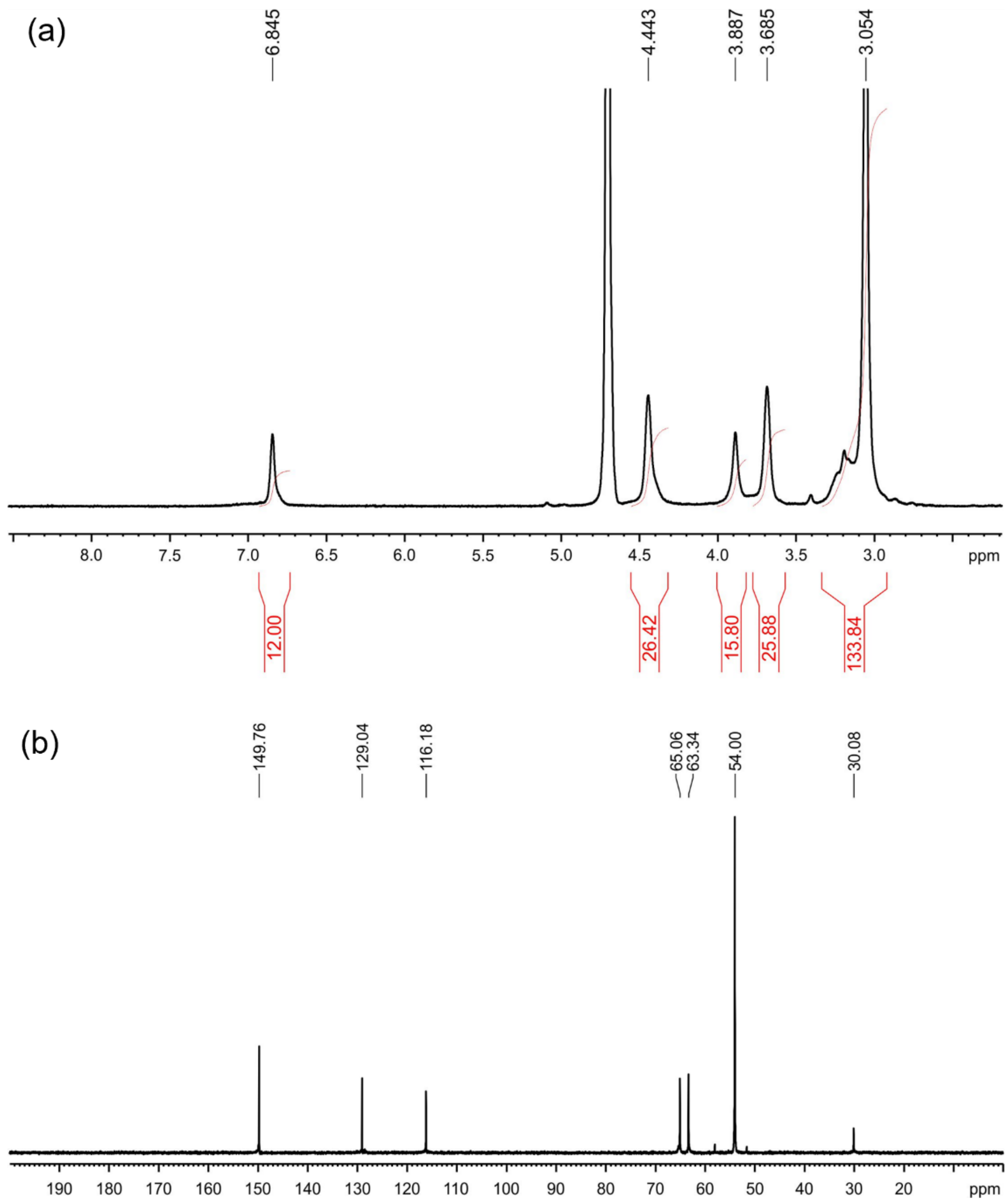

Figure S3. (a) ^{1}H NMR, (b) ^{13}C NMR of **P12+** in D_2O at 298 K.

(a)

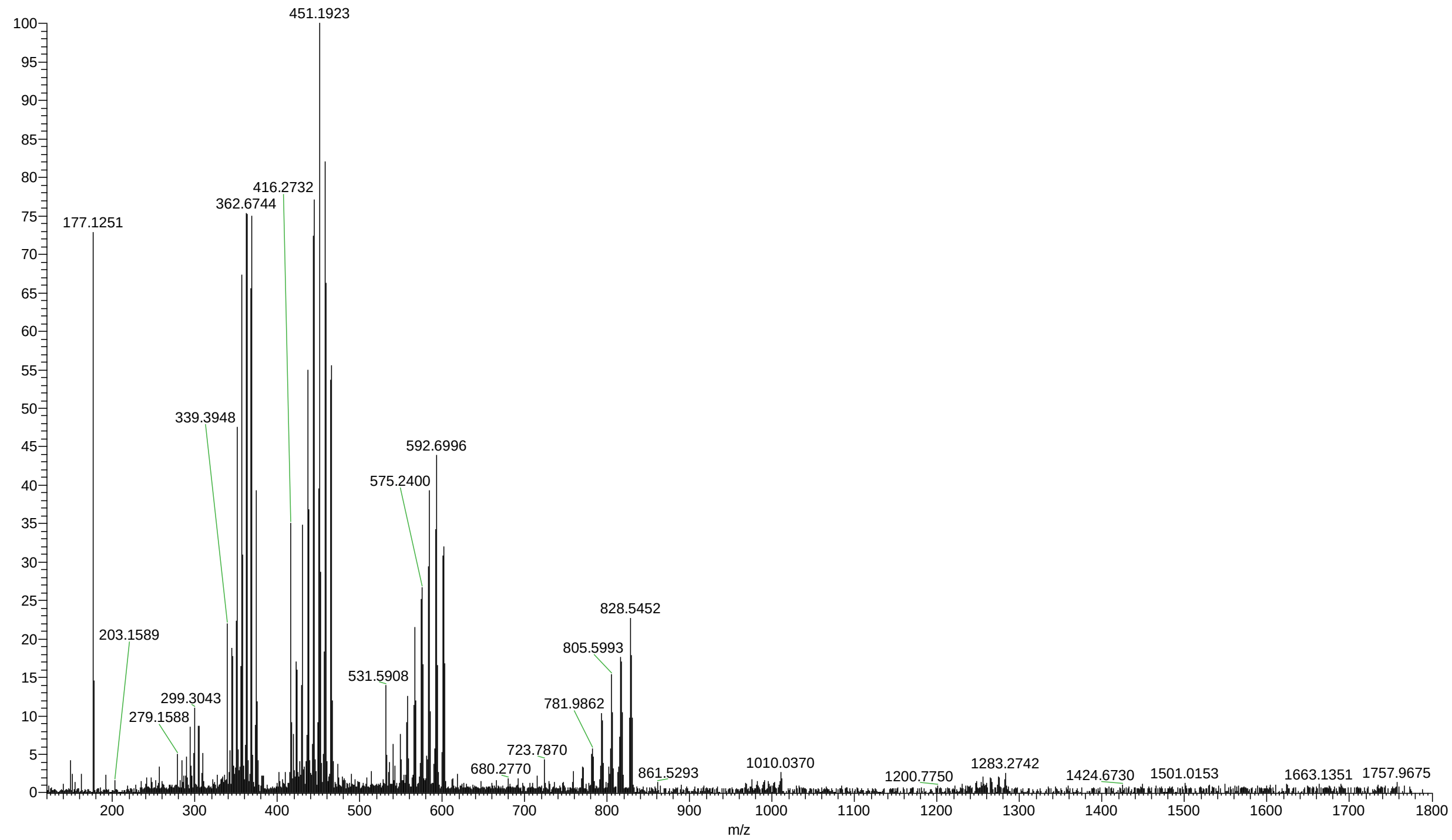

(b)

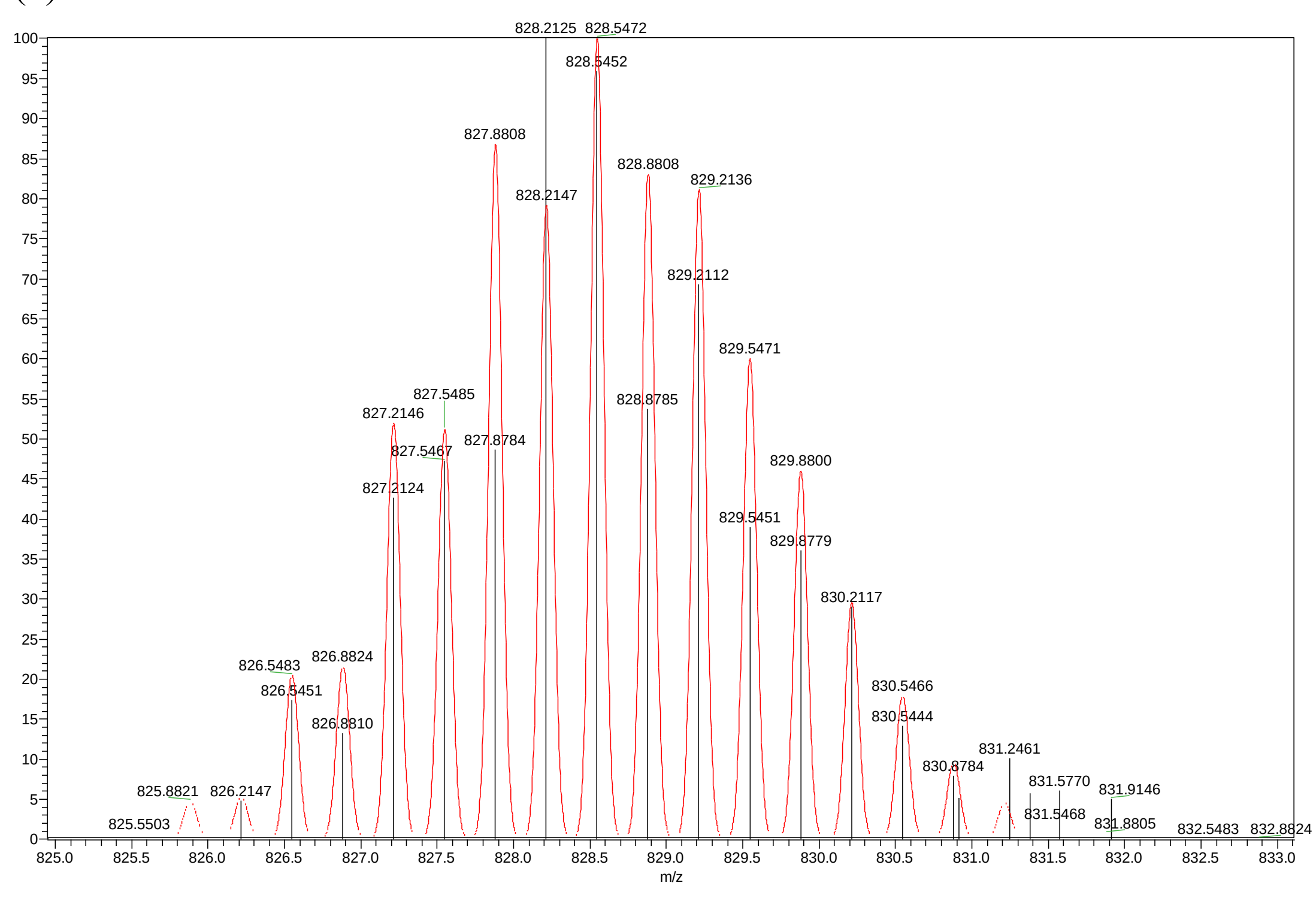

Figure S4. (a) ESI-Mass spectra of **P12+**. (b) $[M\text{-}3Br]^{3+}$ with simulated spectrum.

III. Isothermal Titration Calorimetry (ITC)

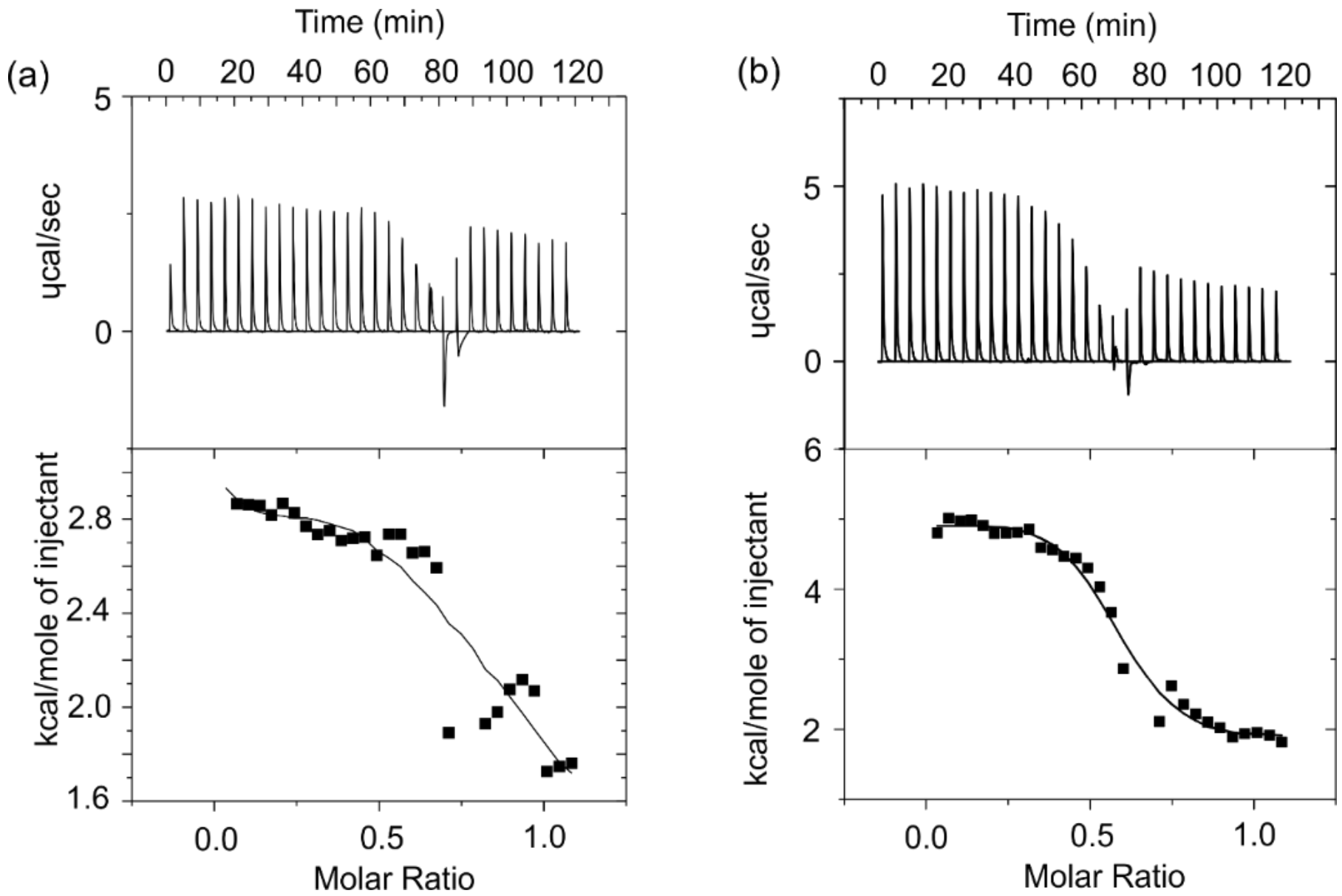

Figure S5. ITC binding data for the titration of Heparin into (a) **P10+** and (b) **P12+**, in TRIS-NaCl buffer (10mM Tris, pH 7.4, 150 mM NaCl) at 310 K.

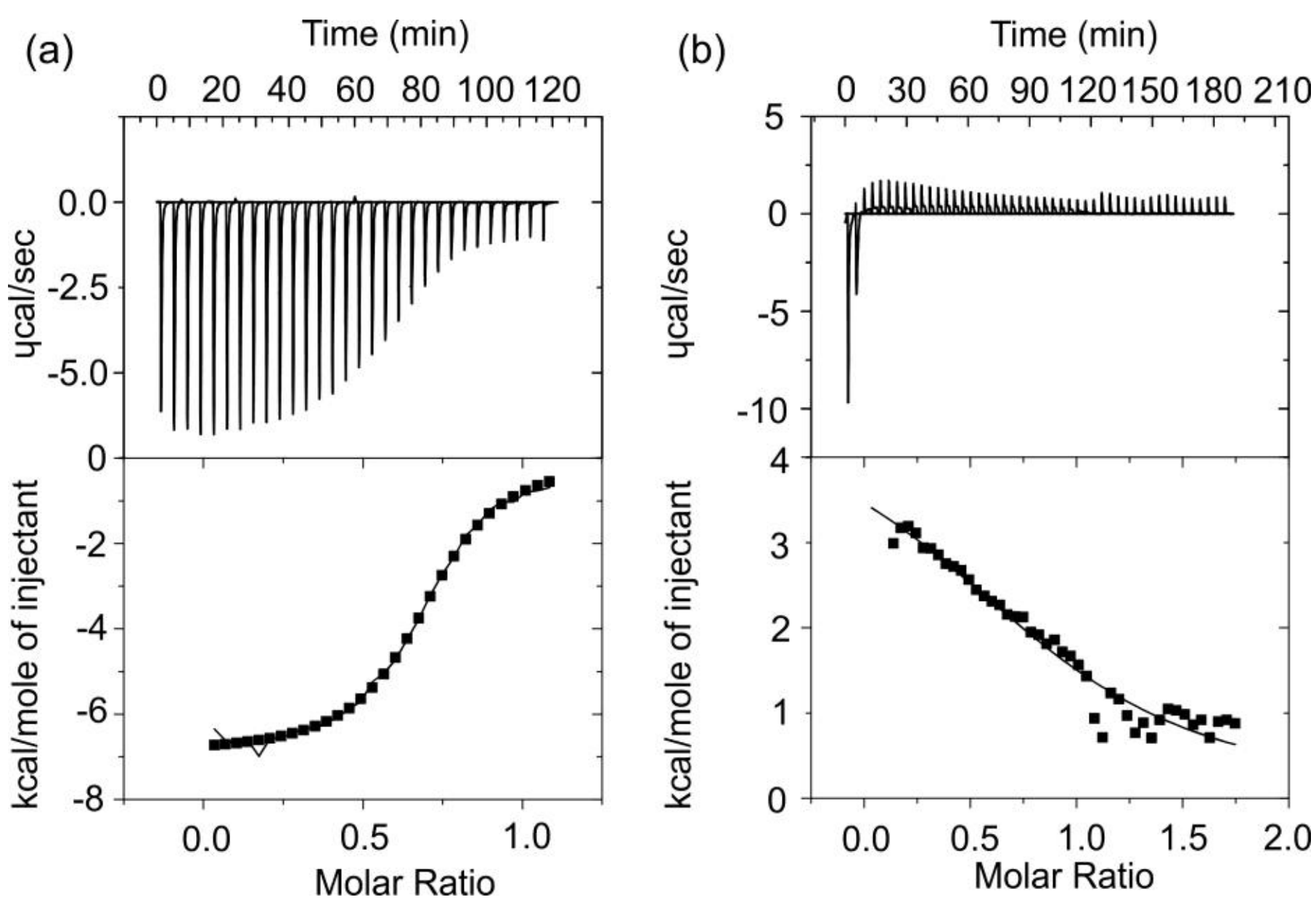

Figure S6. ITC binding data for a) **P10+** and b) **P12+** in TRIS-NaCl buffer (10mM Tris, pH 7.4, 150 mM NaCl) against **MO** at 310K.

IV. Dynamic Light Scattering (DLS)

To assess the size and count rates of binder-heparin complexes, dynamic light scattering experiments were employed in TRIS/NaCl buffer. Equivalences of **P12+** were titrated into 1 mL heparin solution up to 6 mass equivalents. The derived count rate and hydrodynamic diameters were derived from triplicate experiments. This was repeated for **P10+** and protamine sulfate, respectively. Measurements were done using the Malvern Zetasizer instrument.

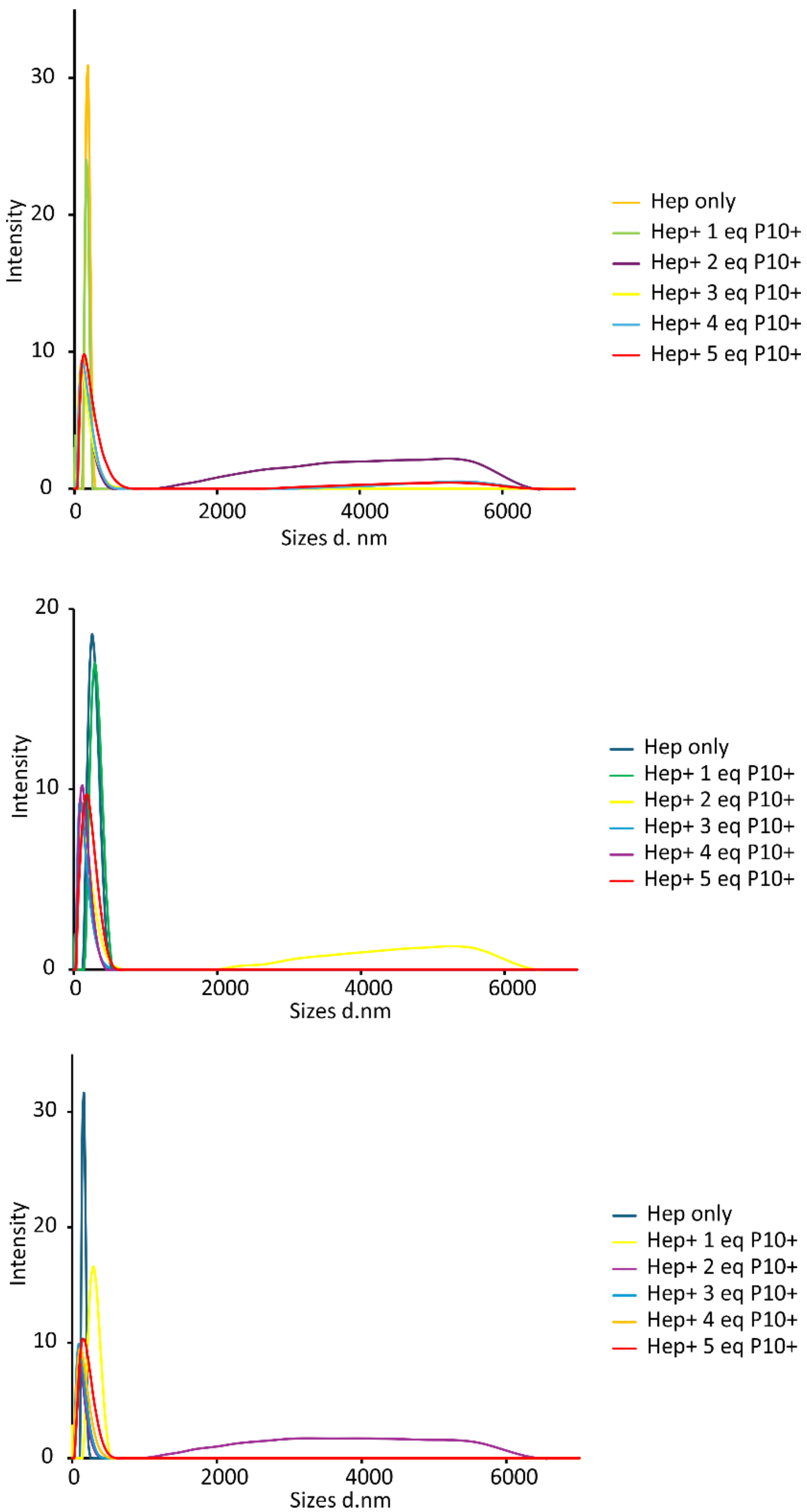

Figure S7. A plot illustrating the size distributions of Heparin in a mixture with different equivalents of **P10+** in a 10 mM Tris buffer.

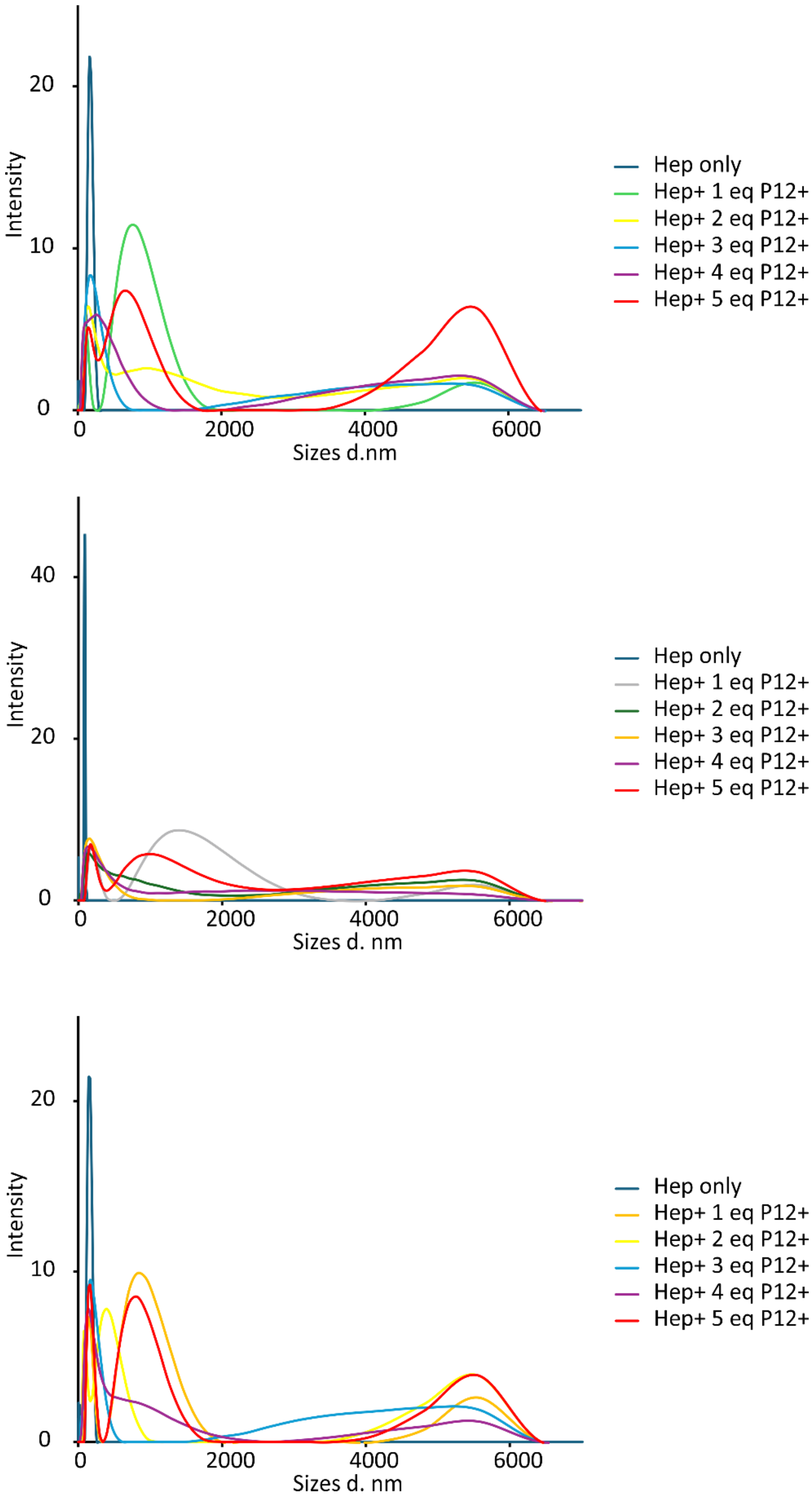

Figure S8. A plot illustrating the size distributions of Heparin in a mixture with different equivalents of **P12+** in a 10 mM Tris buffer.

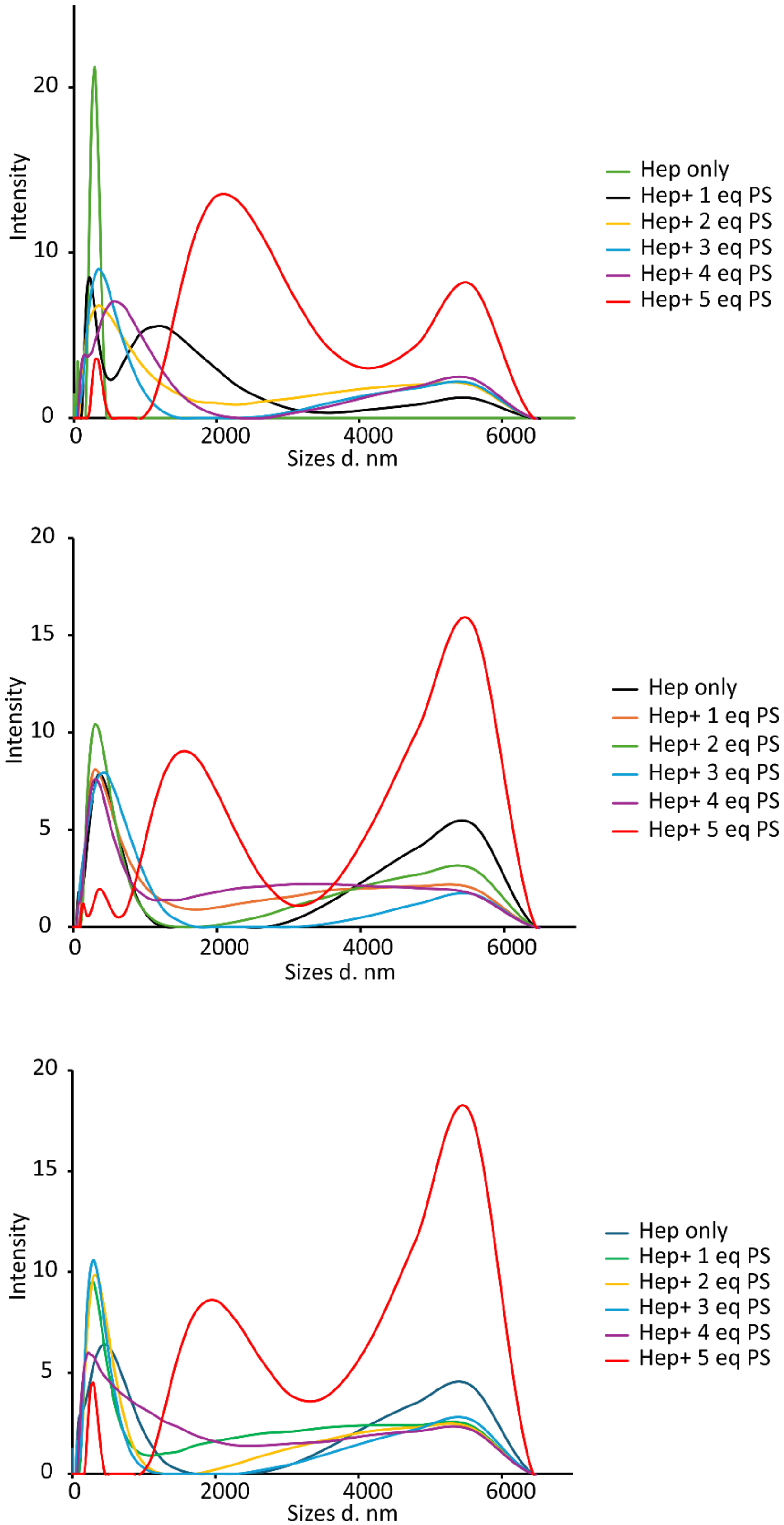

Figure S9. A plot illustrating the size distributions of Heparin in a mixture with different equivalents of **PS** in a 10 mM Tris buffer.

V. Anti-Xa

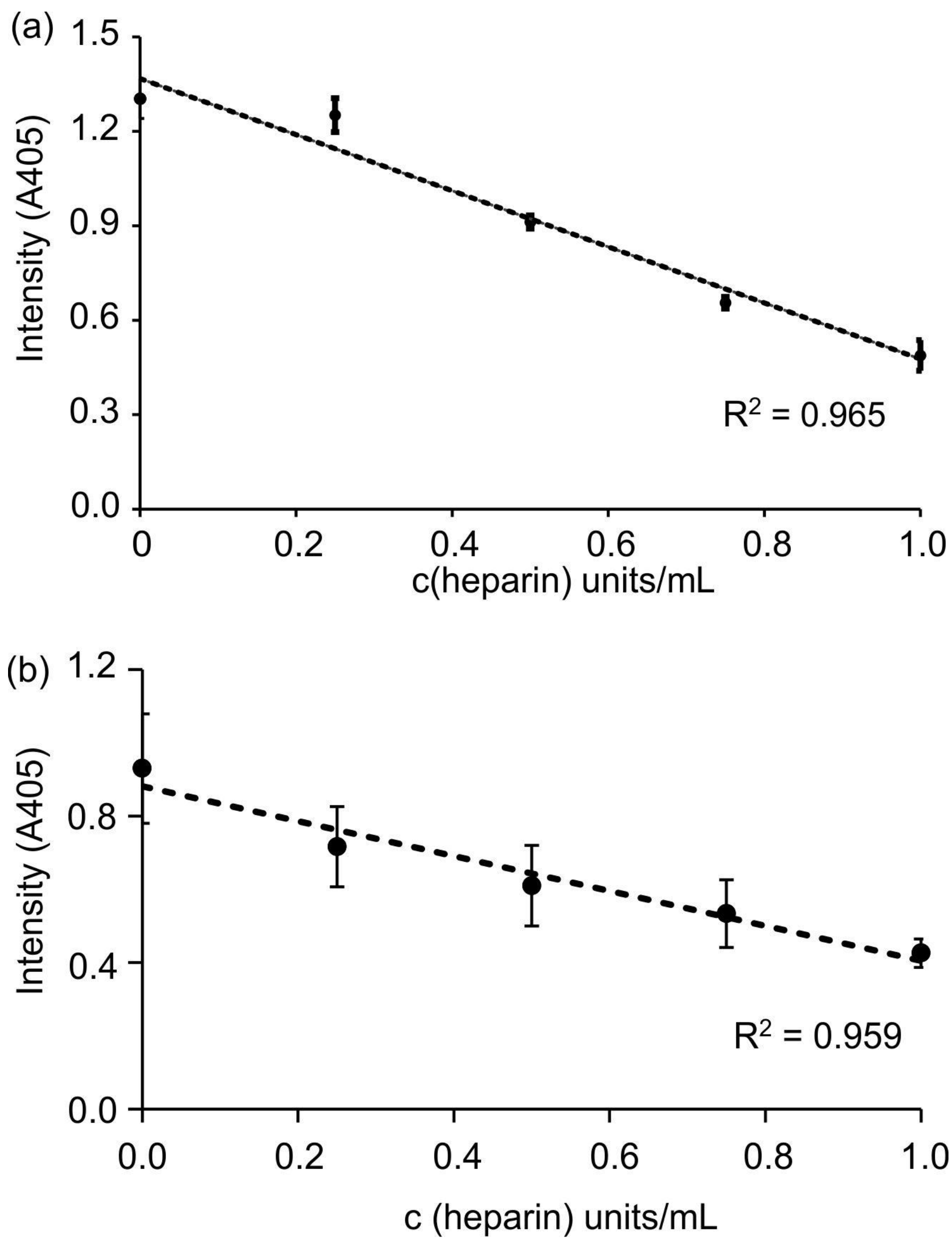

Figure S10: Calibration curve for anti-Xa assay at 405 nm in plasma for **P12+** against: (a) Unfractionated heparin, and (b) Low molecular weight heparin.

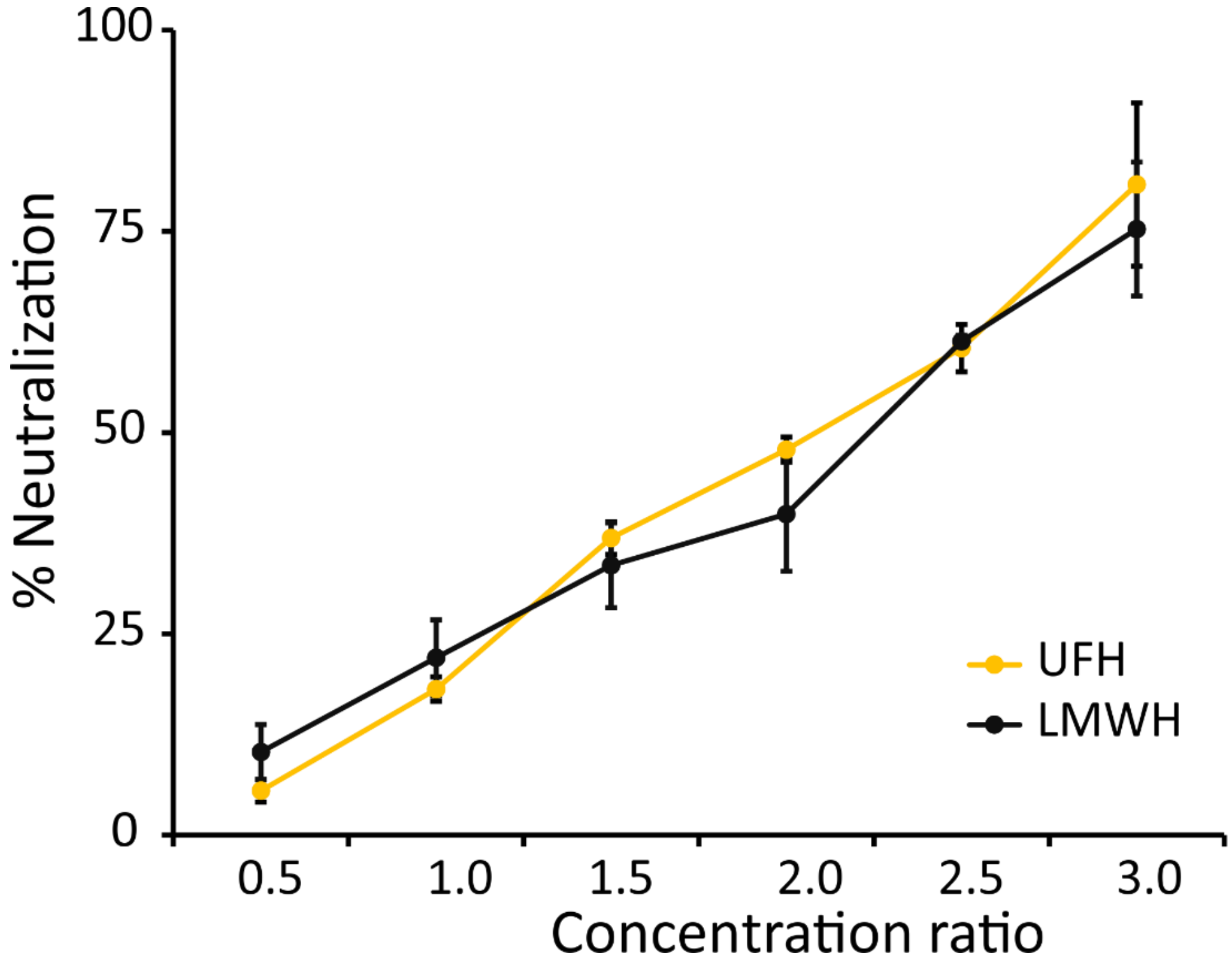

Figure S11. The anti-Xa assay of neutralizer (**P10+**) against unfractionated (UFH) and low-molecular-weight heparins (LMWH).

VI. Confocal Fluorescence Microscopy (CFM)

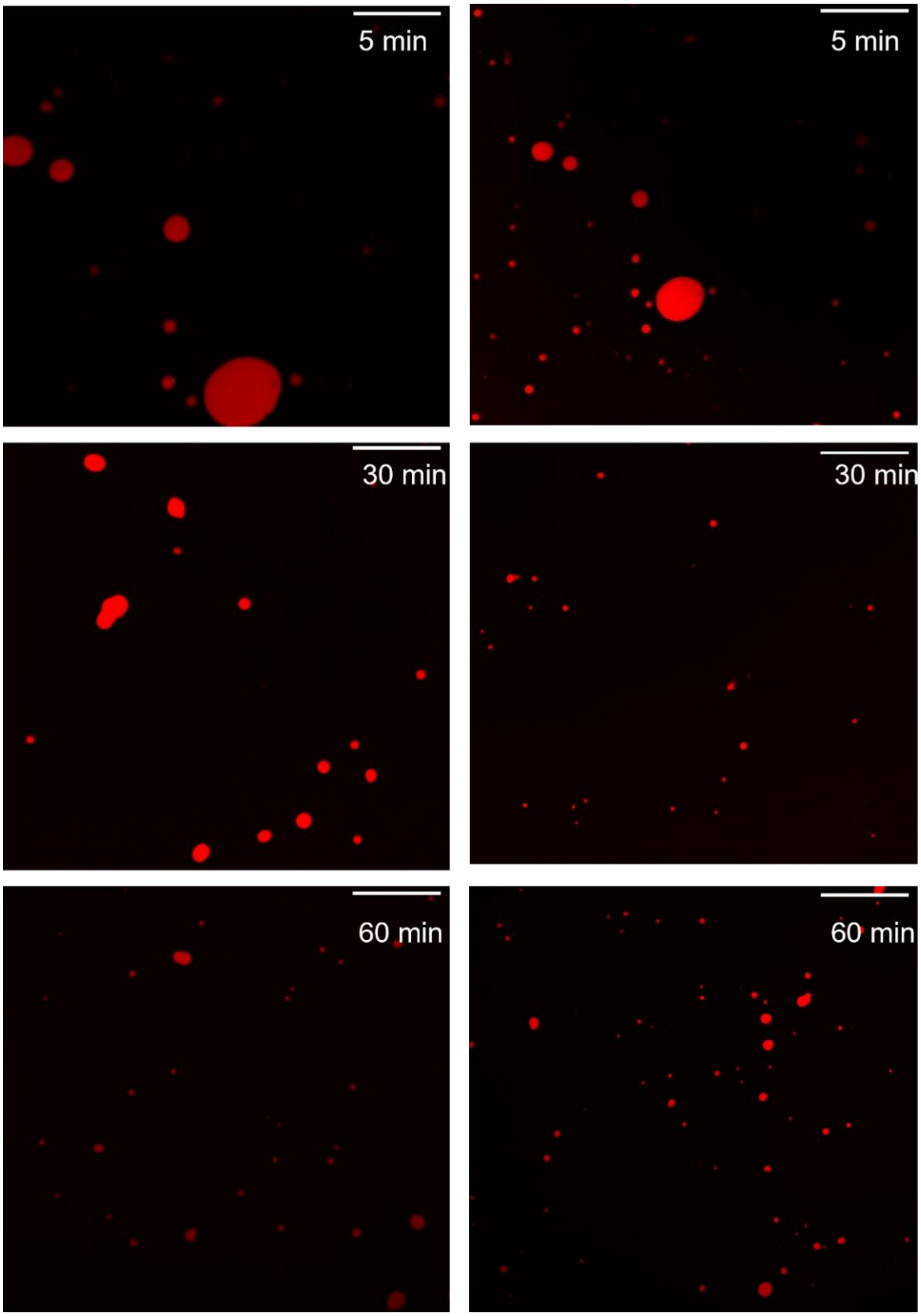

Figure S12. Time-dependent heparin uptake by **P12+** captured on confocal fluorescence microscope using rhodamine B at 561 nm.

VII. UV-Vis and Fluorescence Spectroscopy

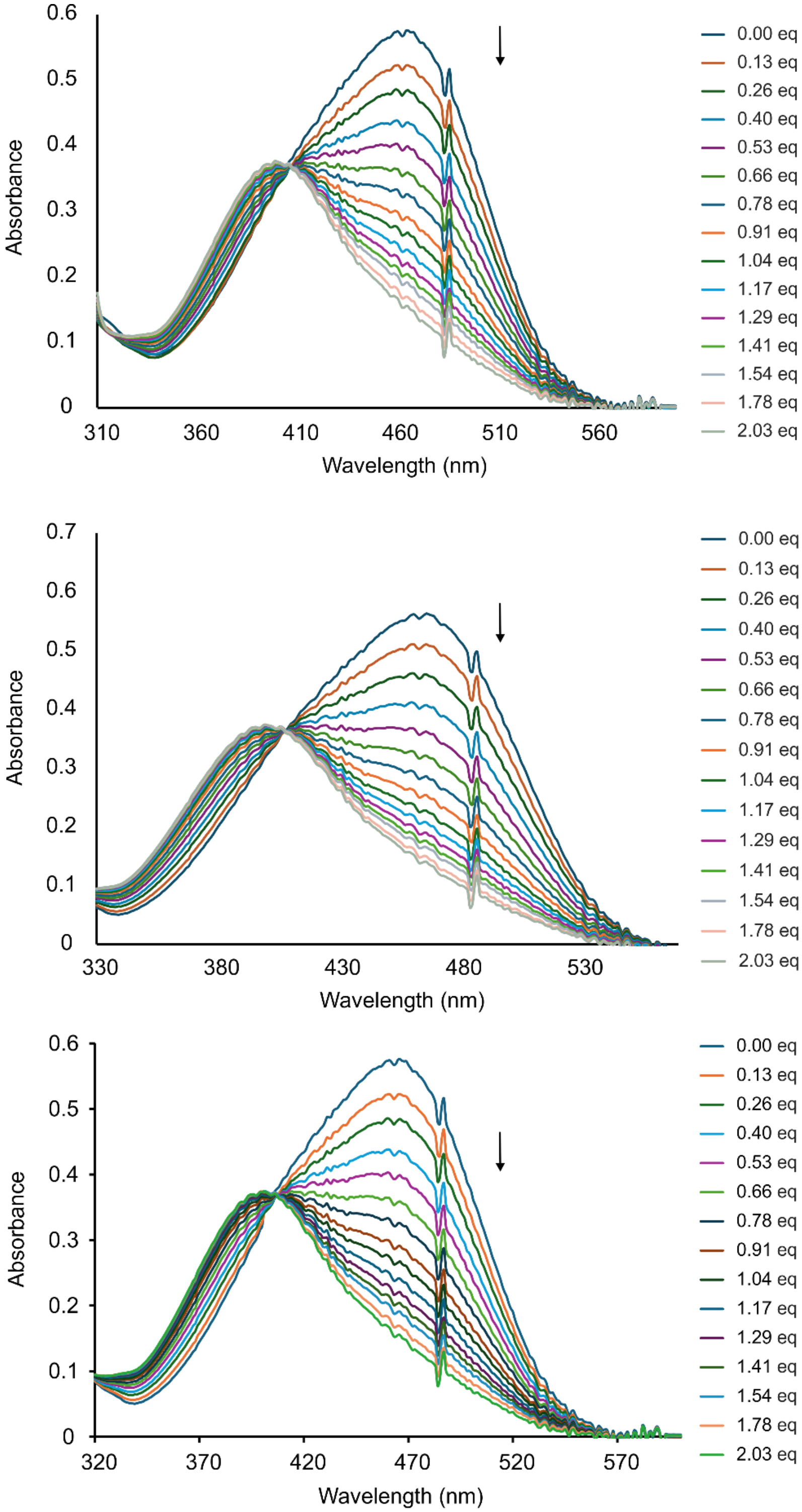

Figure S13. UV absorption spectra of the titration of **P10+** into **MO** in TRIS buffer (10 mM, pH 7.4, 150 mM NaCl)

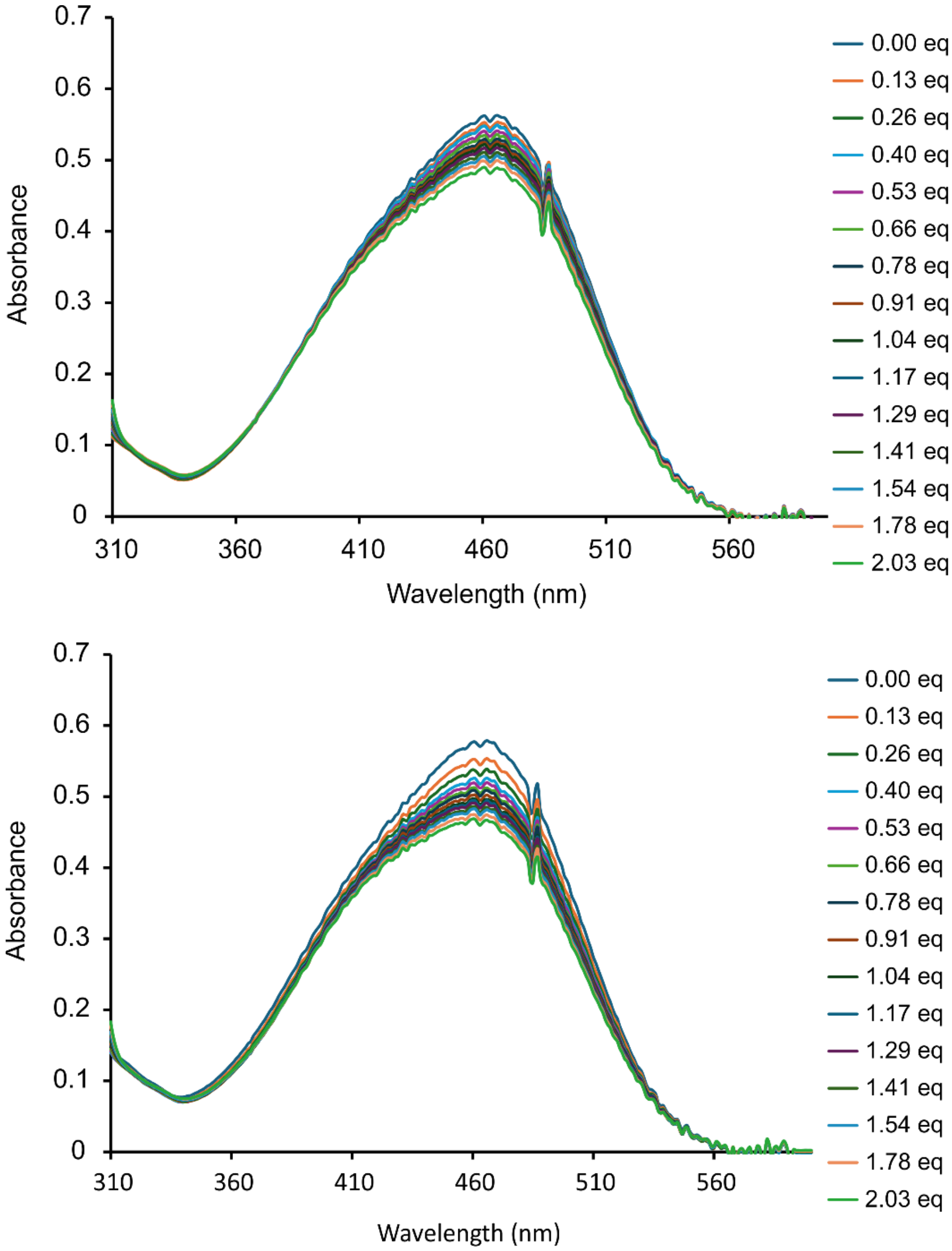

Figure S14. UV absorption spectra of the titration of **P12+** into **MO** in TRIS buffer (10 mM, pH 7.4, 150 mM NaCl). **P12+** vs **MO** at lower equivalents.

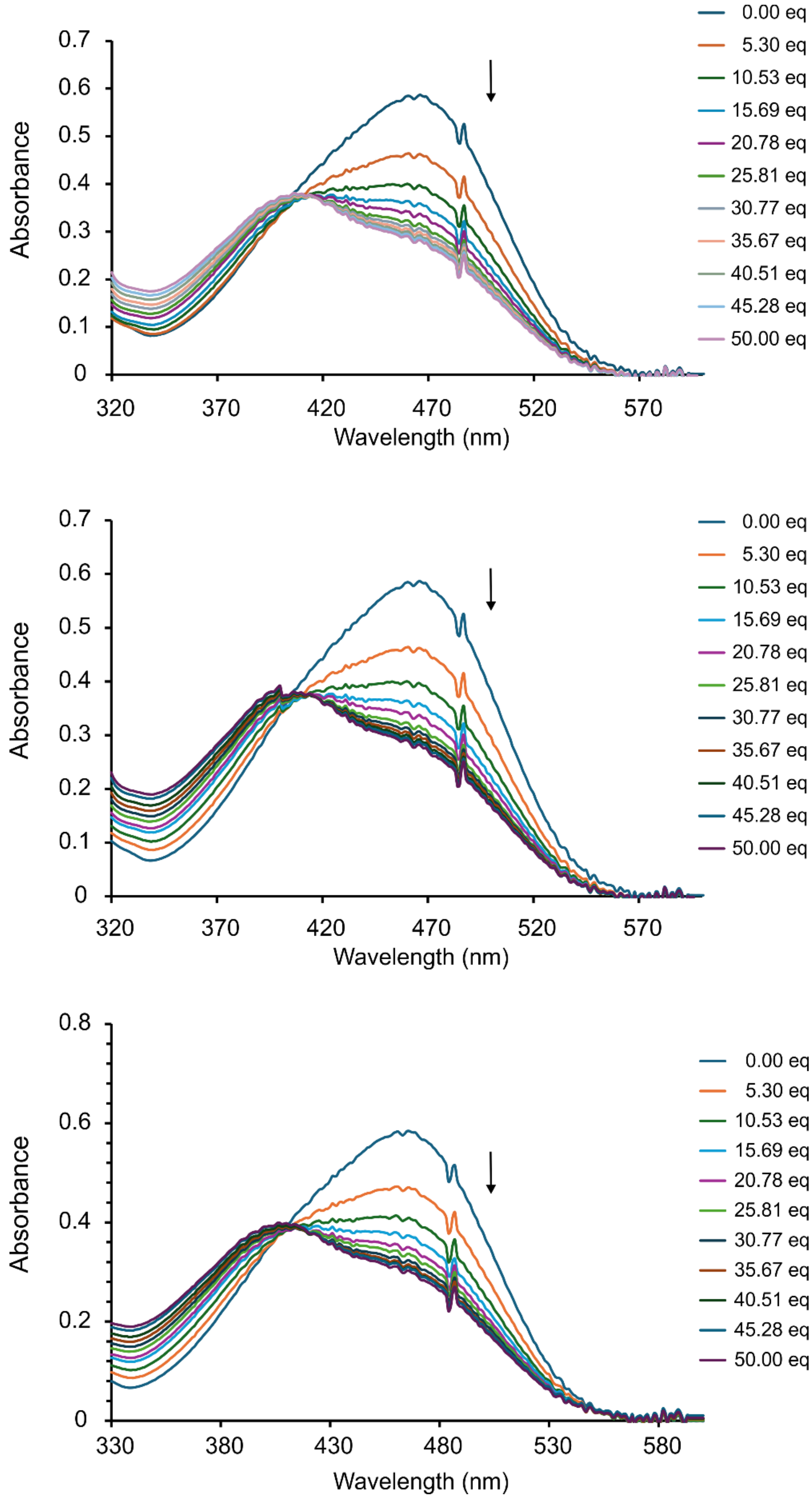

Figure S15. UV absorption spectra of the titration of **P12+** into **MO** in TRIS buffer (10 mM, pH 7.4, 150 mM NaCl). **P12+** vs **MO** at higher equivalents.

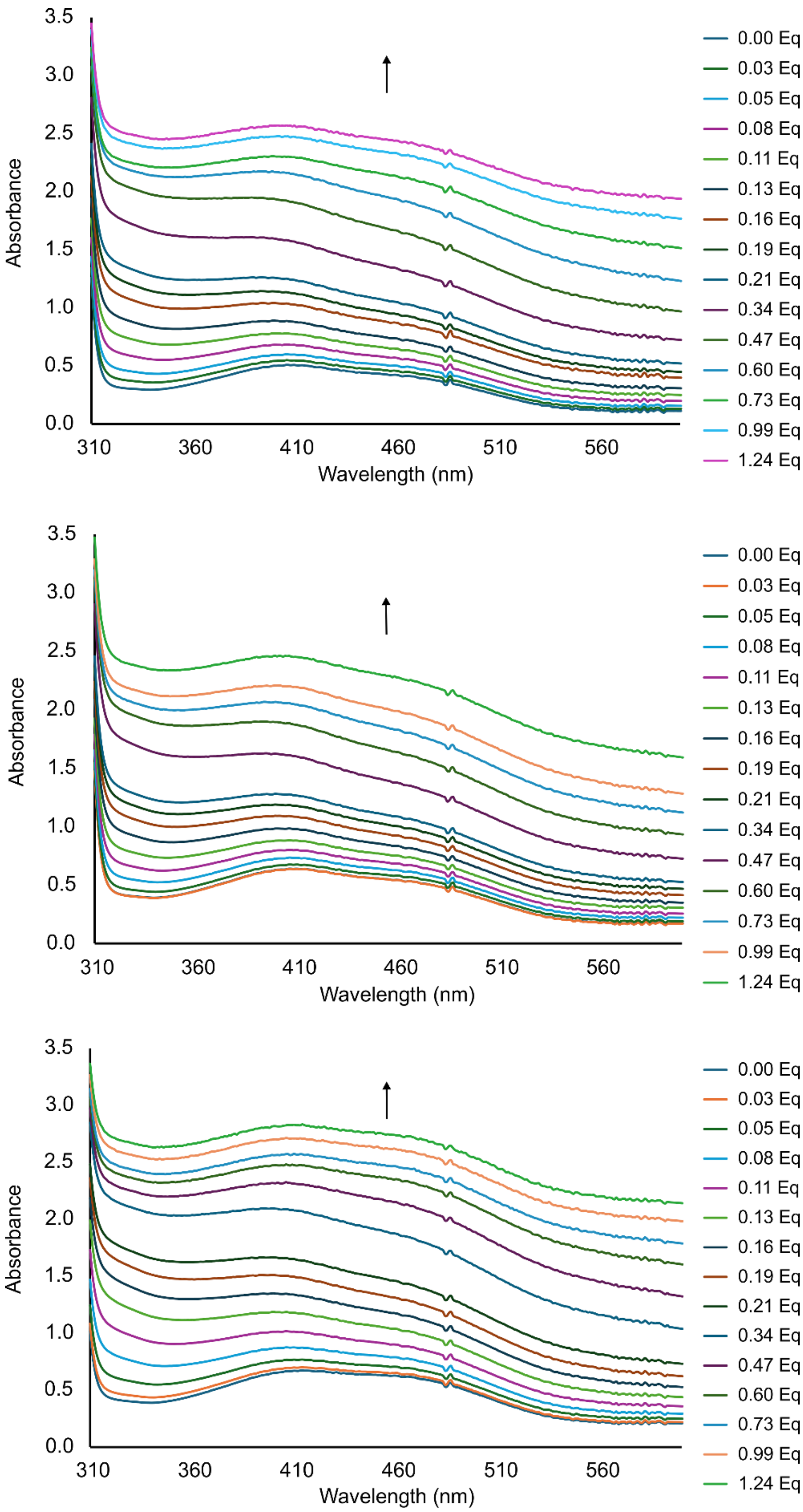

Figure S16. UV absorption spectra of the titration of Heparin into a 35:1 mixture of **P12+•MO** in TRIS-NaCl buffer (10mM Tris, pH 7.4, 150 mM NaCl)

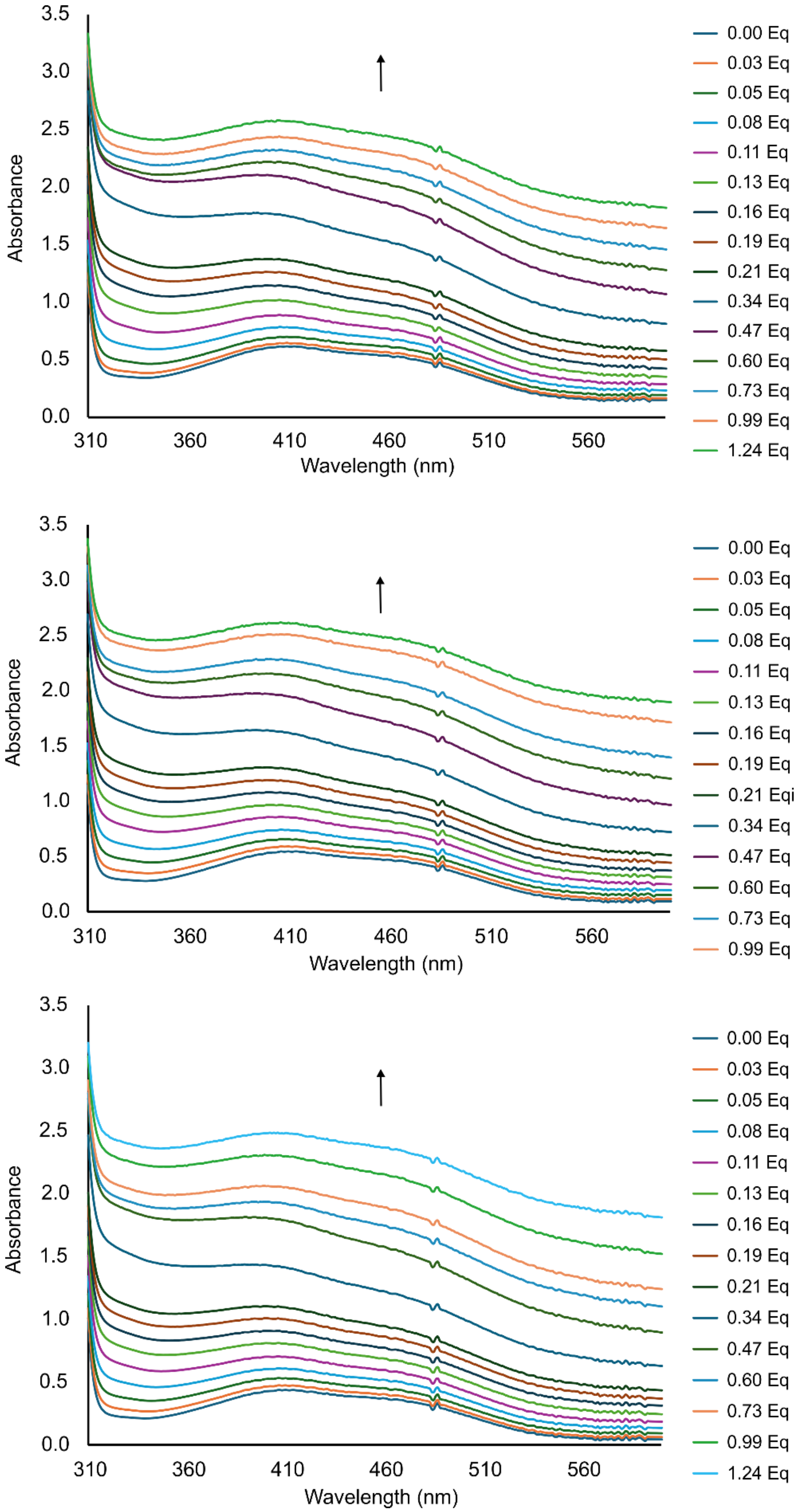

Figure S17. UV absorption spectra of the titration of Heparin into a 30:1 mixture of **P12+•MO** in TRIS-NaCl buffer (10mM Tris, pH 7.4, 150 mM NaCl)

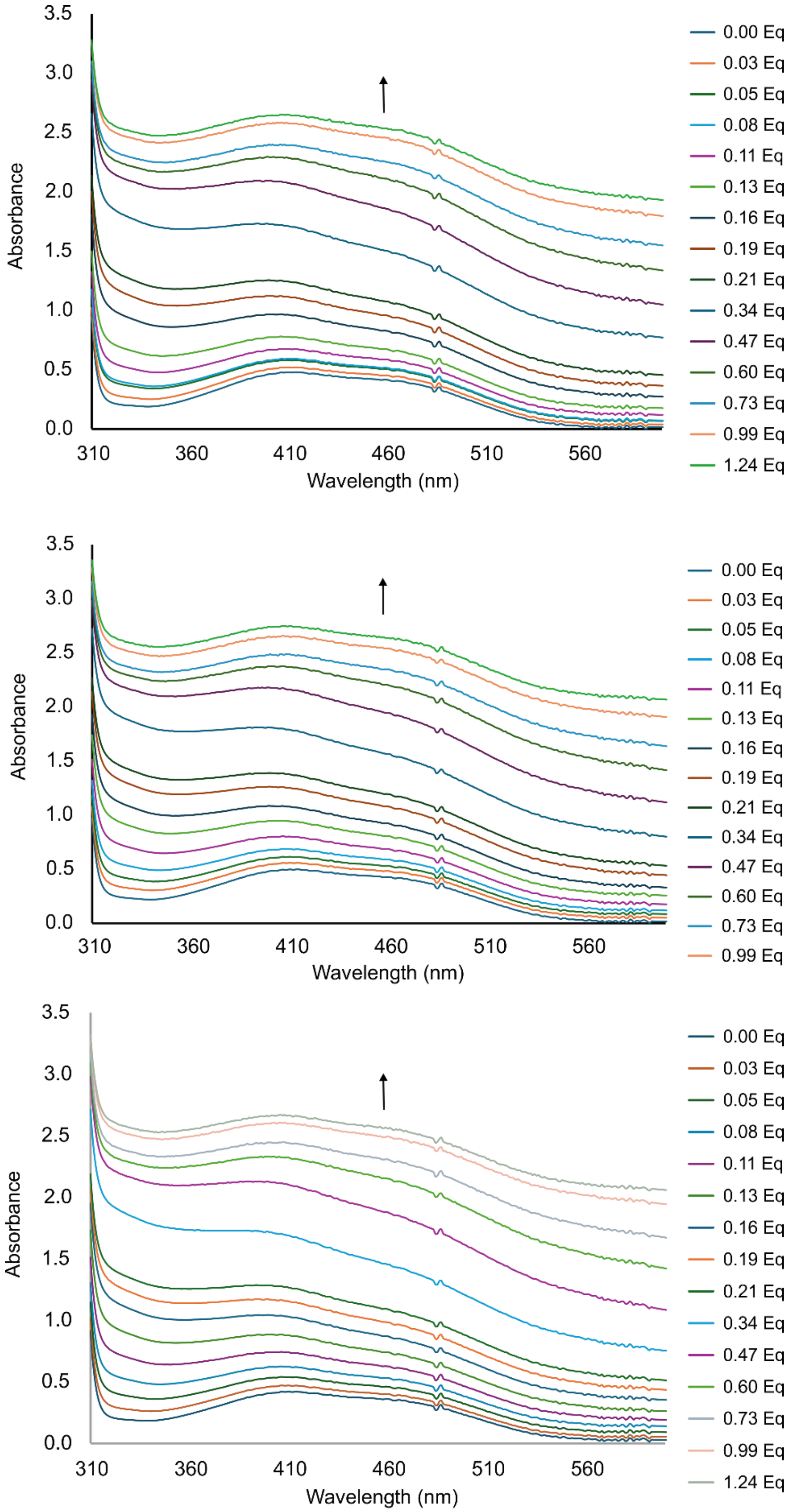

Figure S18. UV absorption spectra of the titration of Heparin into a 25:1 mixture of **P12+•MO** in TRIS-NaCl buffer (10mM Tris, pH 7.4, 150 mM NaCl)

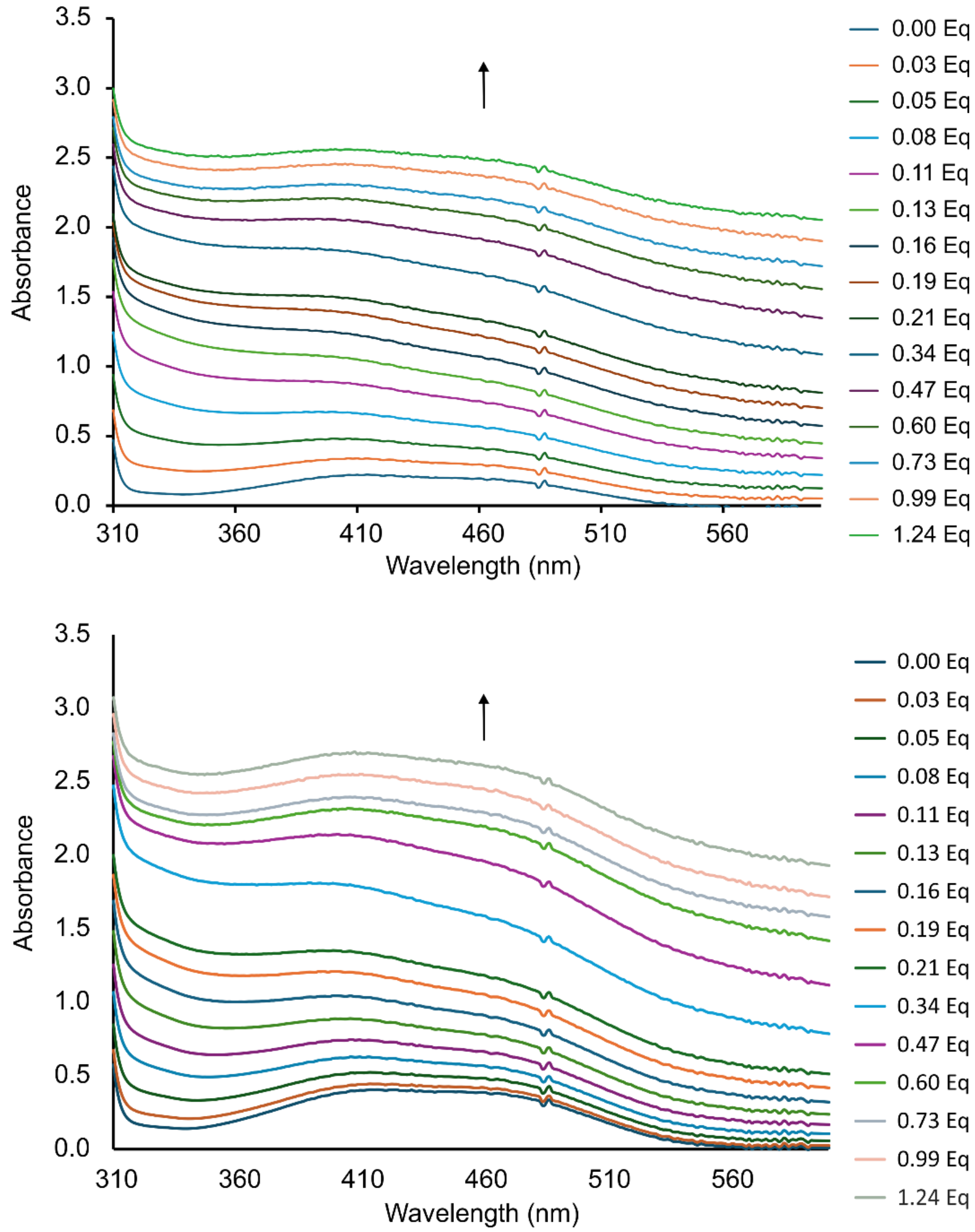

Figure S19. UV absorption spectra of the titration of Heparin into a 15:1 mixture of **P12+•MO** in TRIS-NaCl buffer (10mM Tris, pH 7.4, 150 mM NaCl)

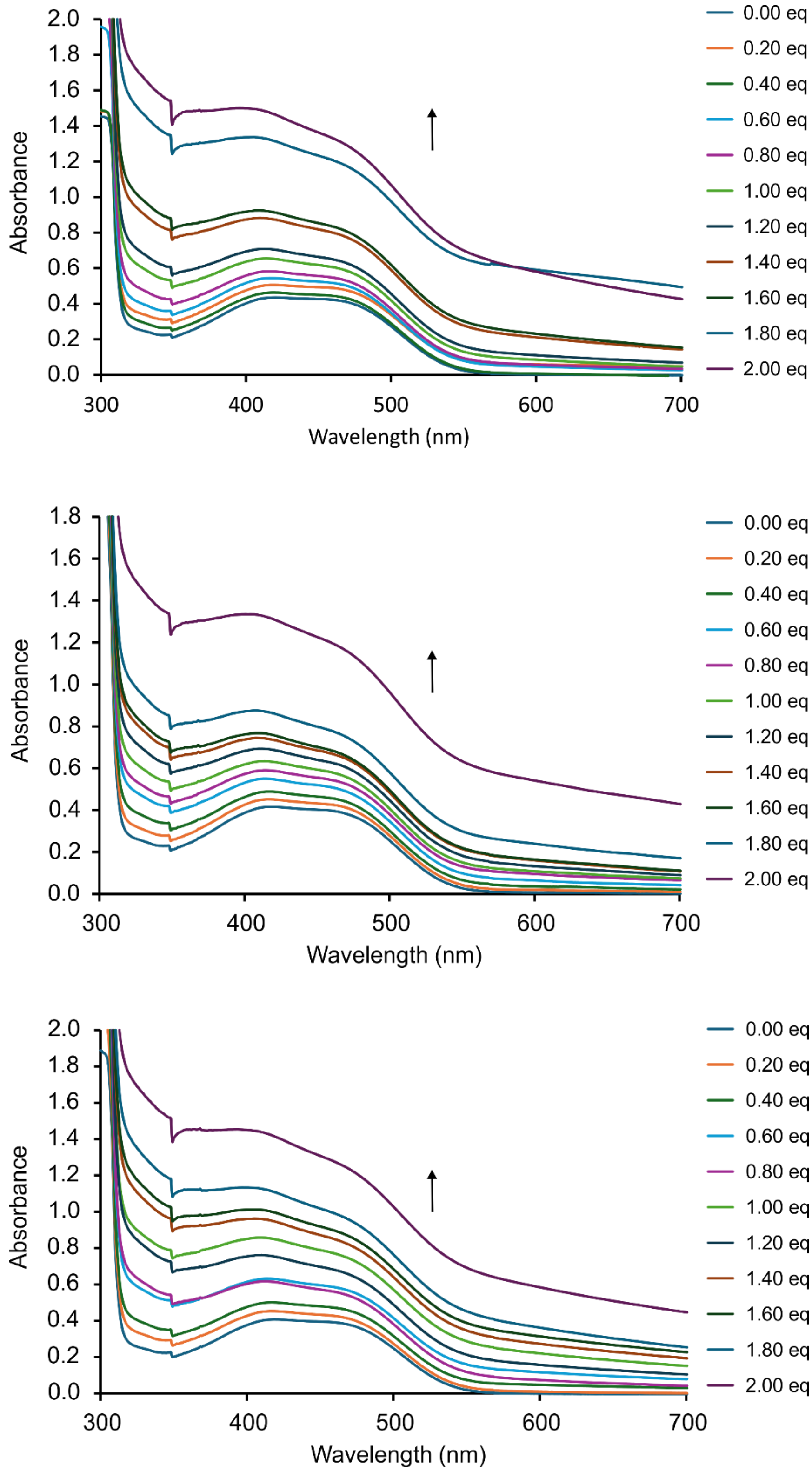

Figure S20. UV absorption spectra of the titration of Heparin into a 25:1 mixture of **P12+•MO** in TRIS-NaCl buffer (10mM Tris, pH 7.4, 150 mM NaCl)

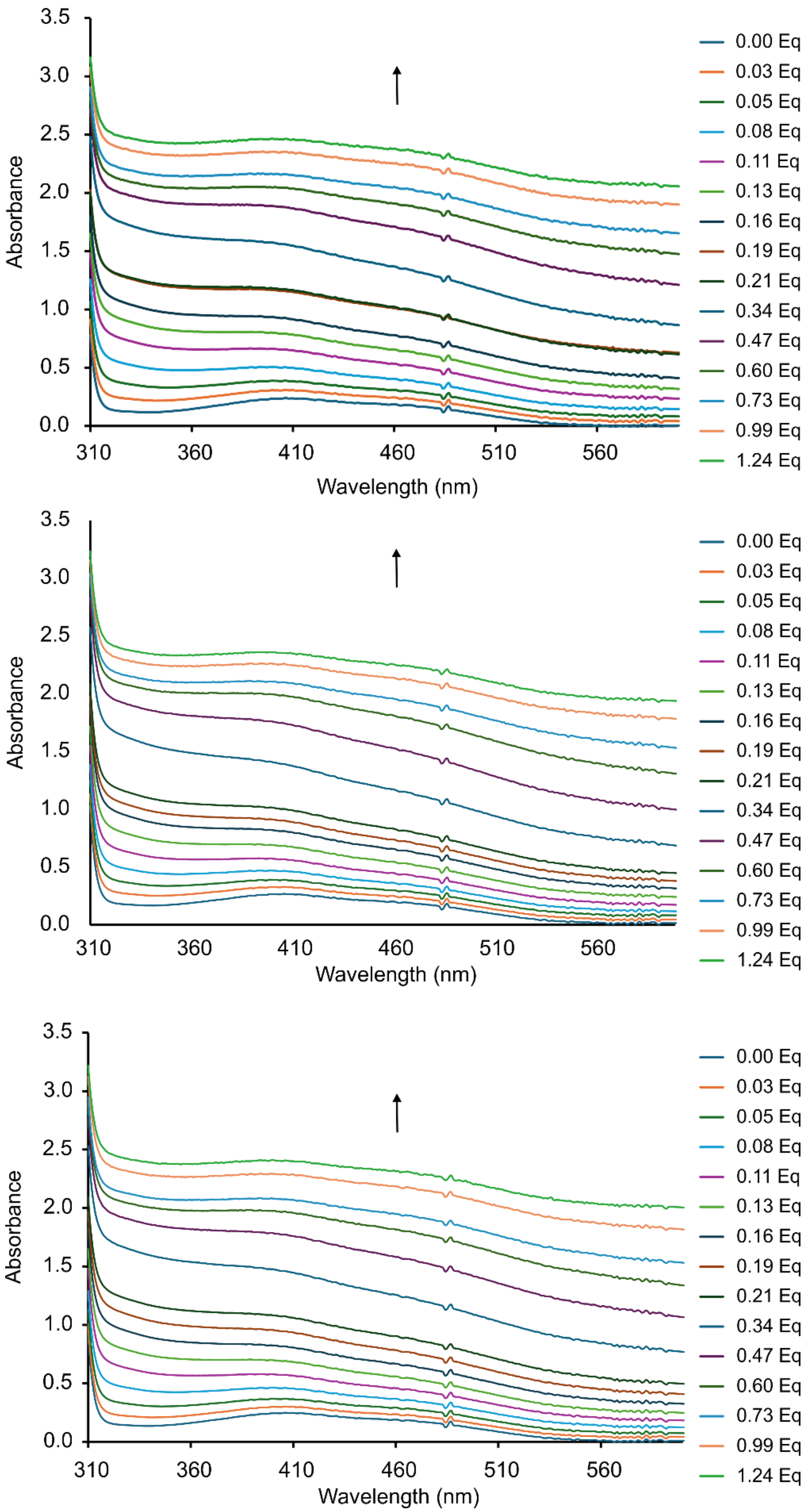

Figure S21. UV absorption spectra of the titration of Heparin into a 25:1 mixture of **P12+•MO** in 5% human plasma (pH 7.4, 150 mM NaCl)

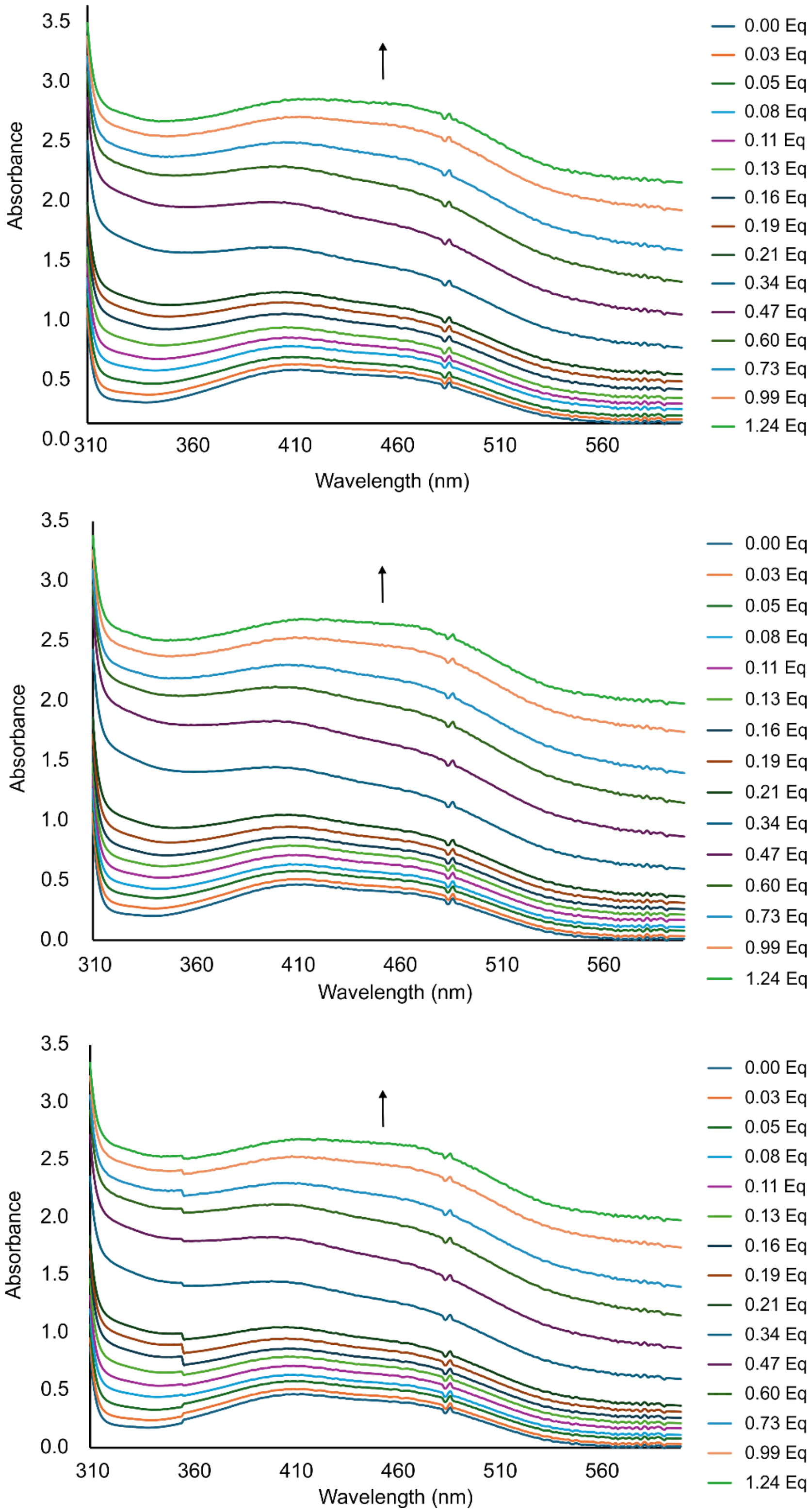

Figure S22. UV absorption spectra of the titration of Heparin into a 25:1 mixture of **P12+•MO** in 50% human plasma (pH 7.4, 150 mM NaCl)

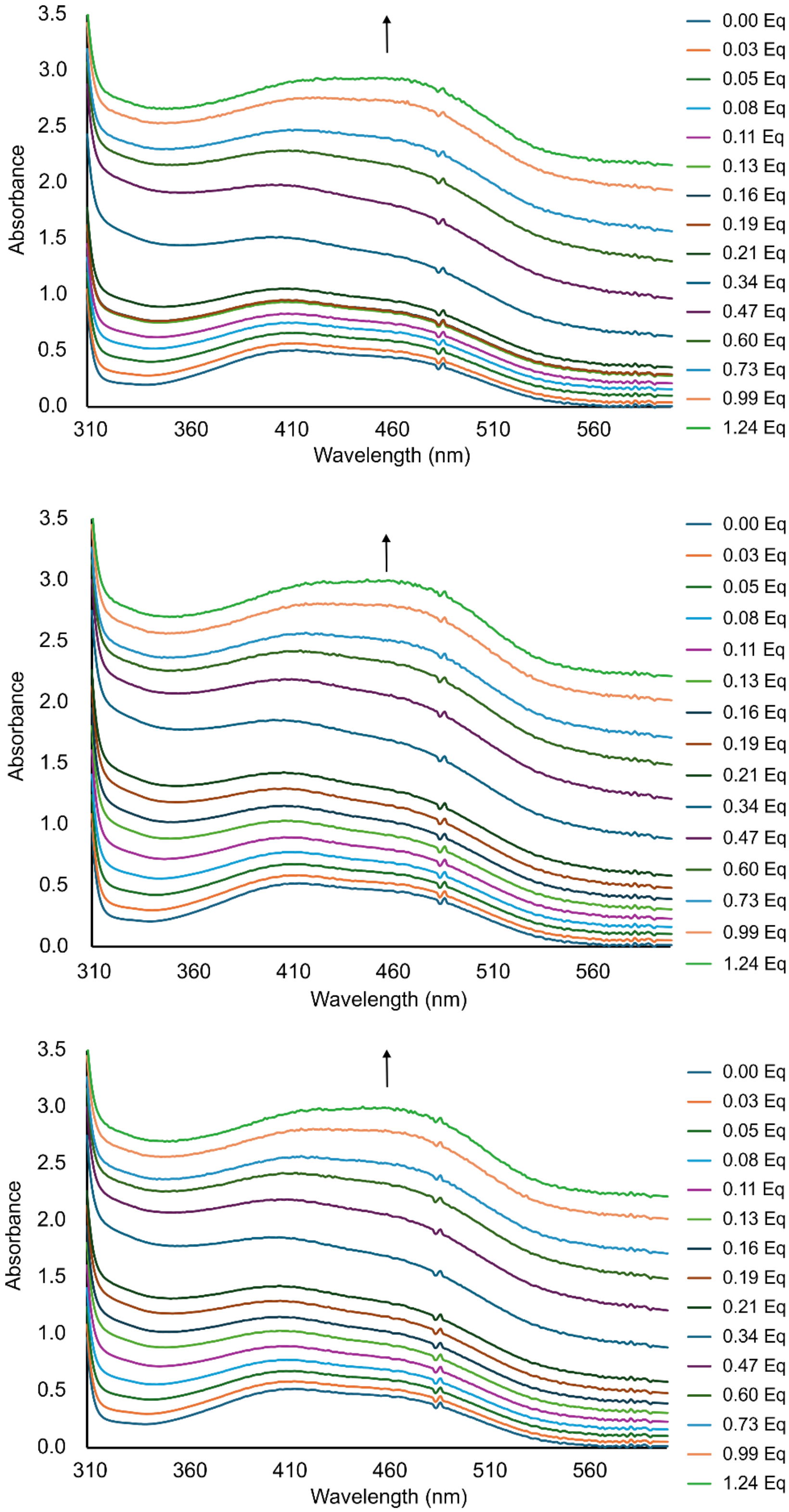

Figure S23. UV absorption spectra of the titration of Heparin into a 25:1 mixture of **P12+•MO** in 100% human plasma (pH 7.4)

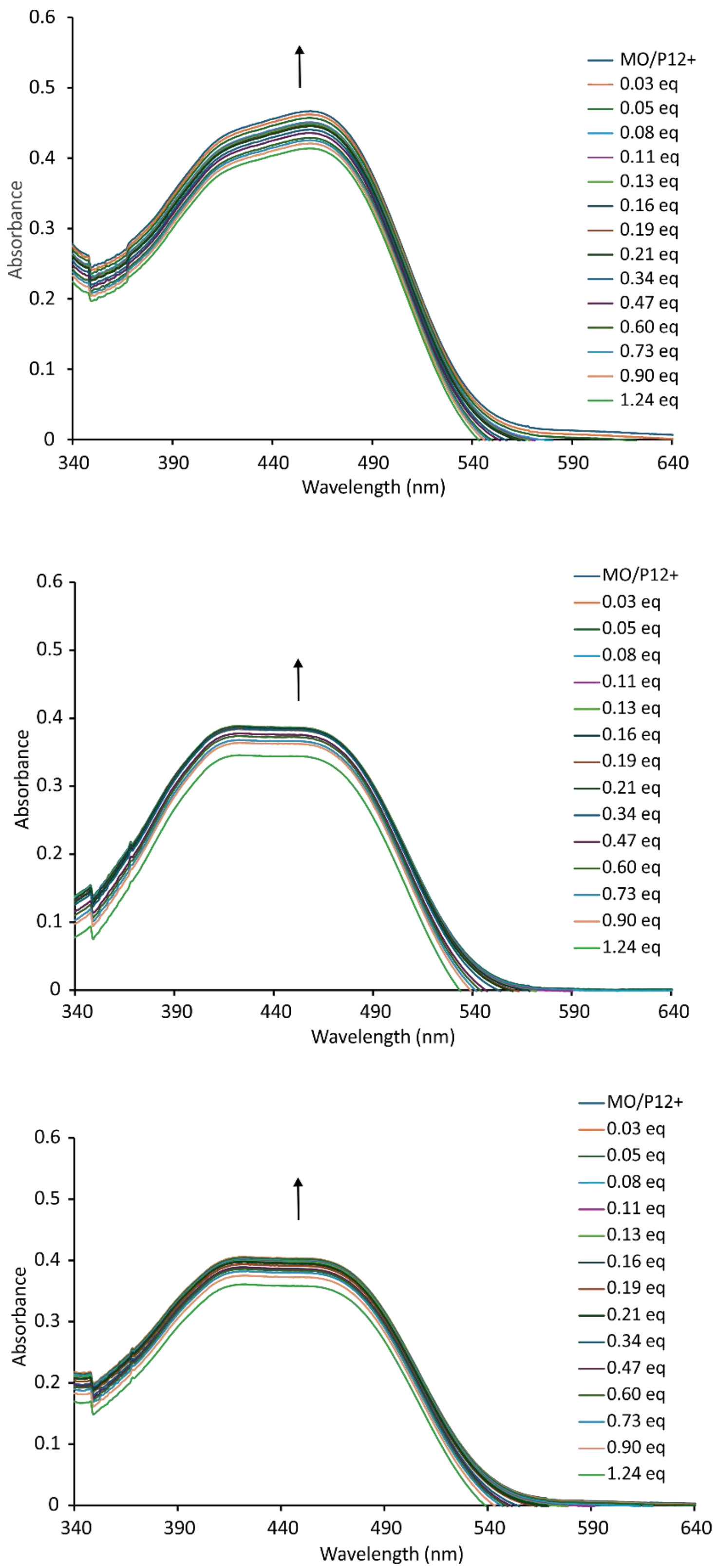

Figure S24. UV absorption spectra of the titration of dextran sulfate into a 25:1 mixture of **P12+•MO** in TRIS-NaCl buffer (10mM Tris, pH 7.4, 150 mM NaCl)

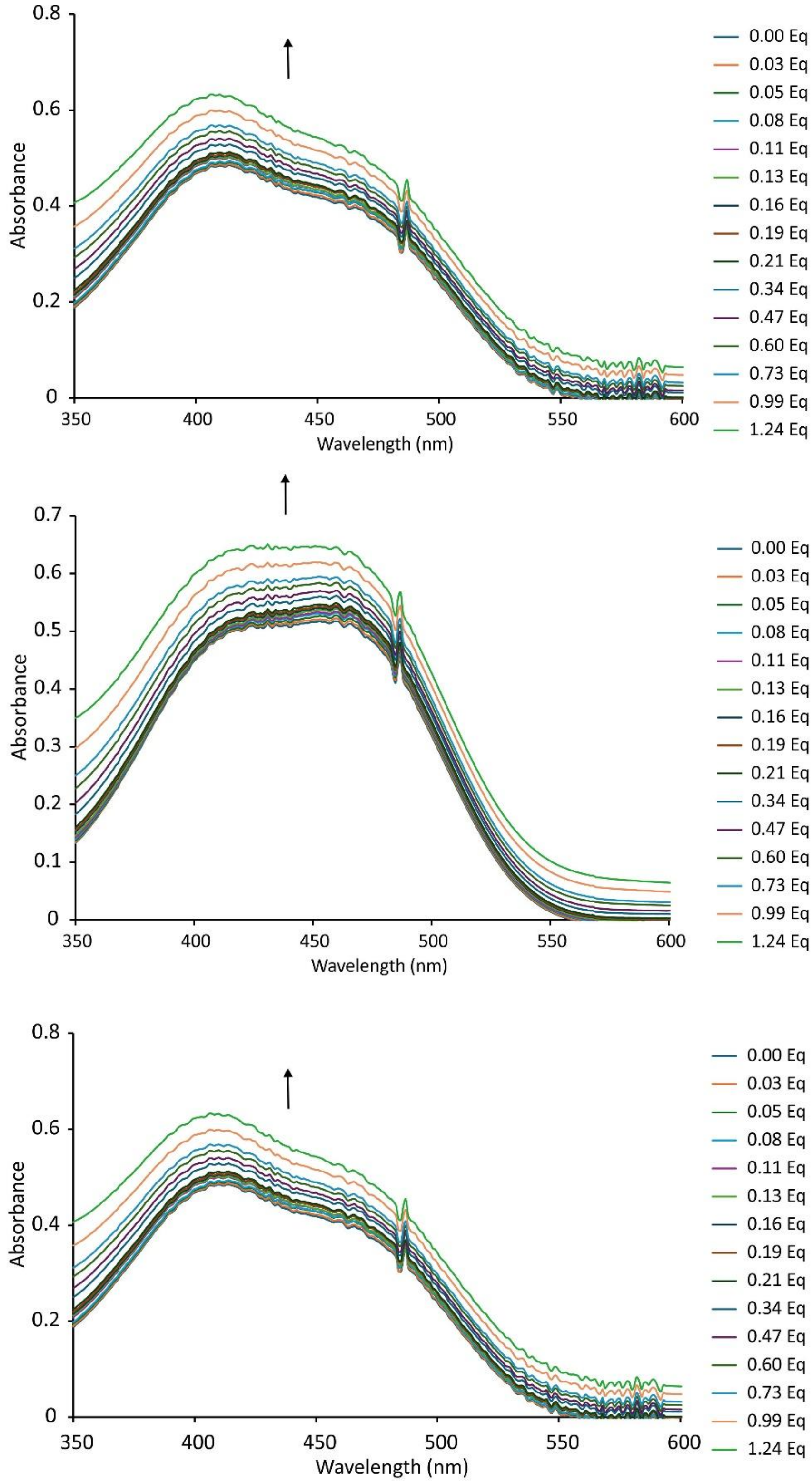

Figure S25. UV absorption spectra of the titration of Chondroitin sulfate into a 25:1 mixture of **P12+•MO** in TRIS-NaCl buffer (10mM Tris, pH 7.4, 150 mM NaCl)

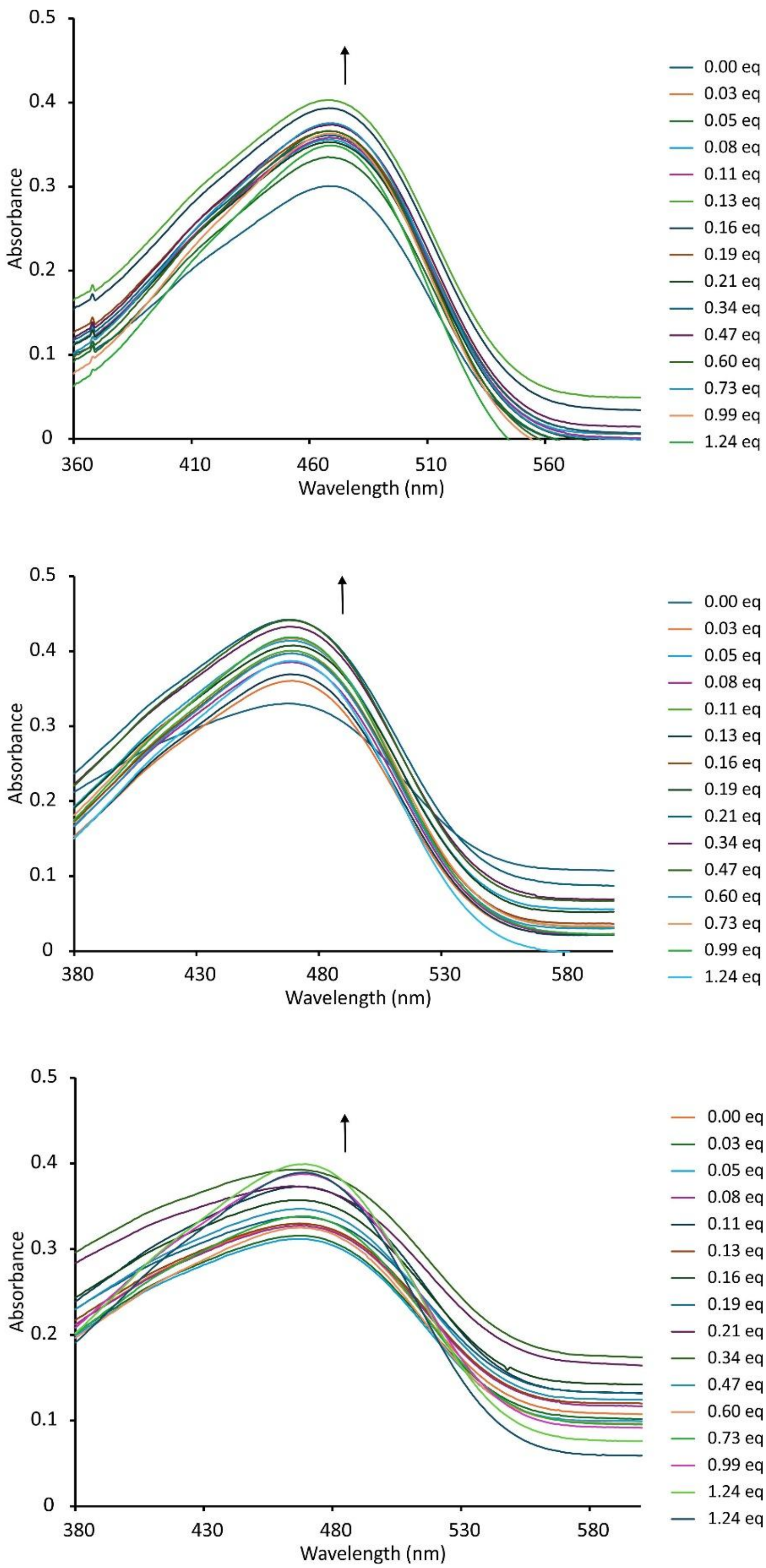

Figure S26. UV absorption spectra of the titration of Hyaluronic acid into a 25:1 mixture of **P12+•MO** in TRIS-NaCl buffer (10mM Tris, pH 7.4, 150 mM NaCl)

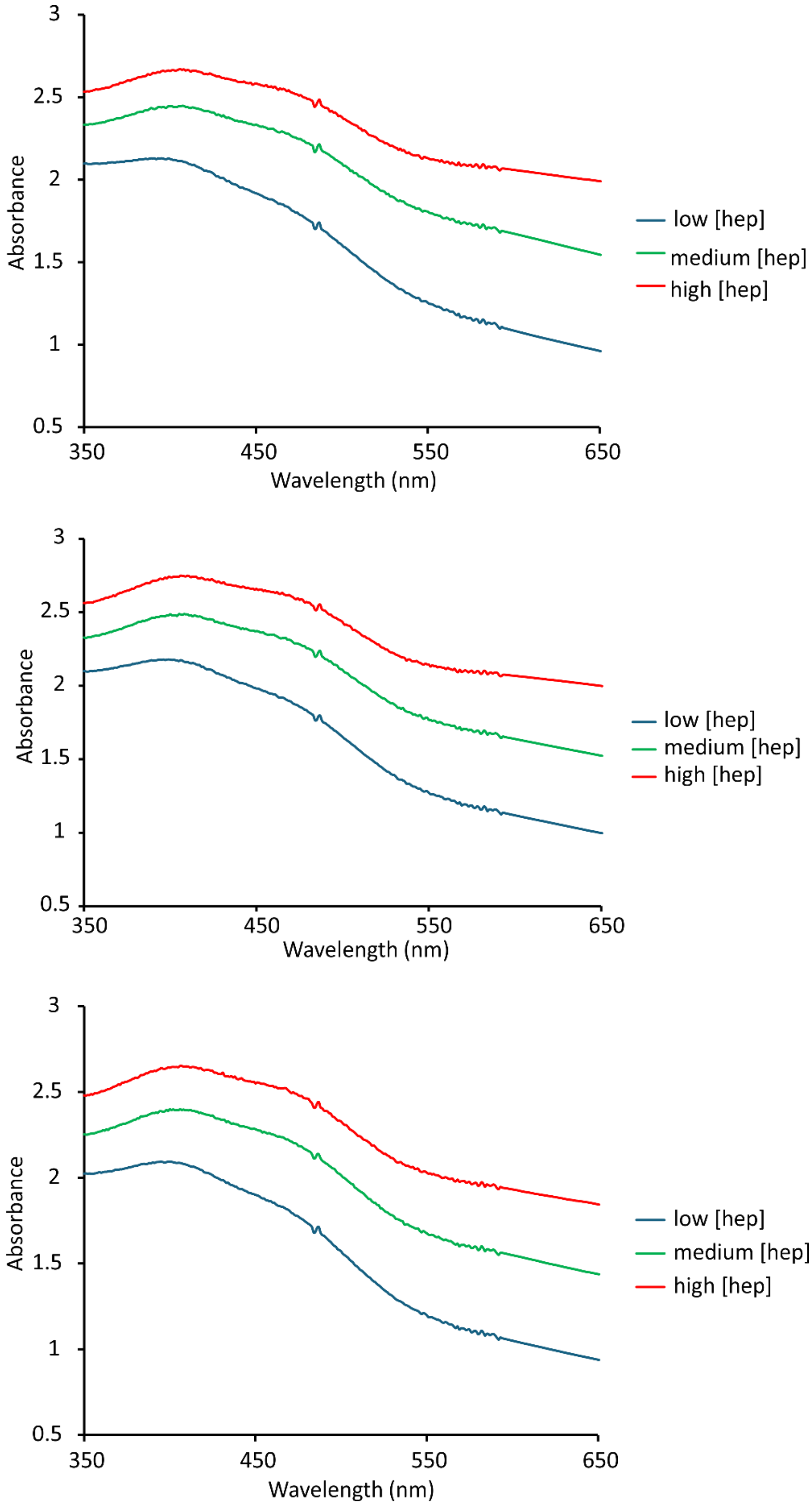

Figure S27. UV absorption spectra showing the spike recovery of the titration of Heparin into a 25:1 mixture of **P12+•MO** in TRIS-NaCl buffer (10mM Tris, pH 7.4, 150 mM NaCl)

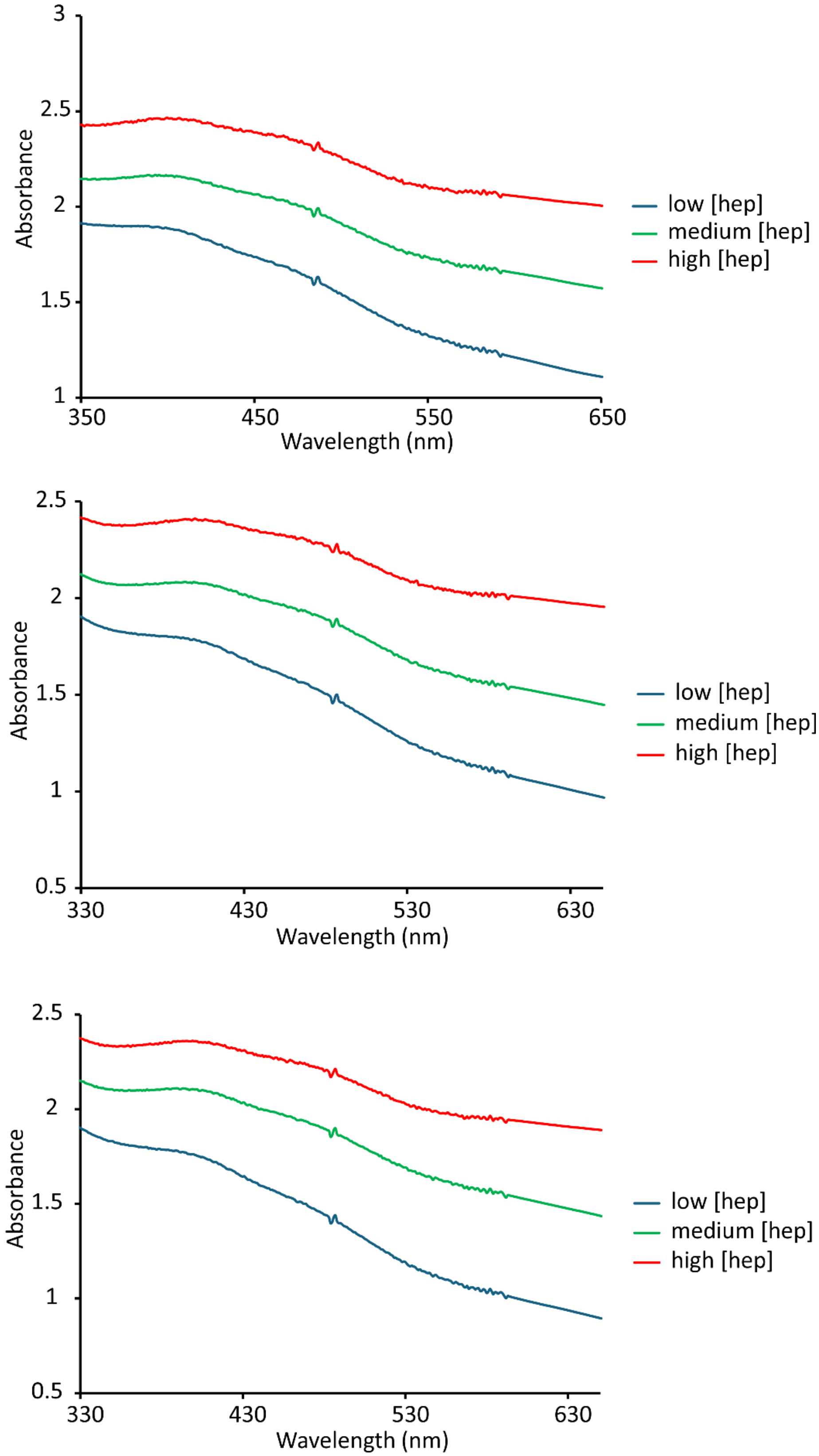

Figure S28. UV absorption spectra showing the spike recovery of the titration of Heparin into a 25:1 mixture of **P12+•MO** in 5% human plasma (pH 7.4)

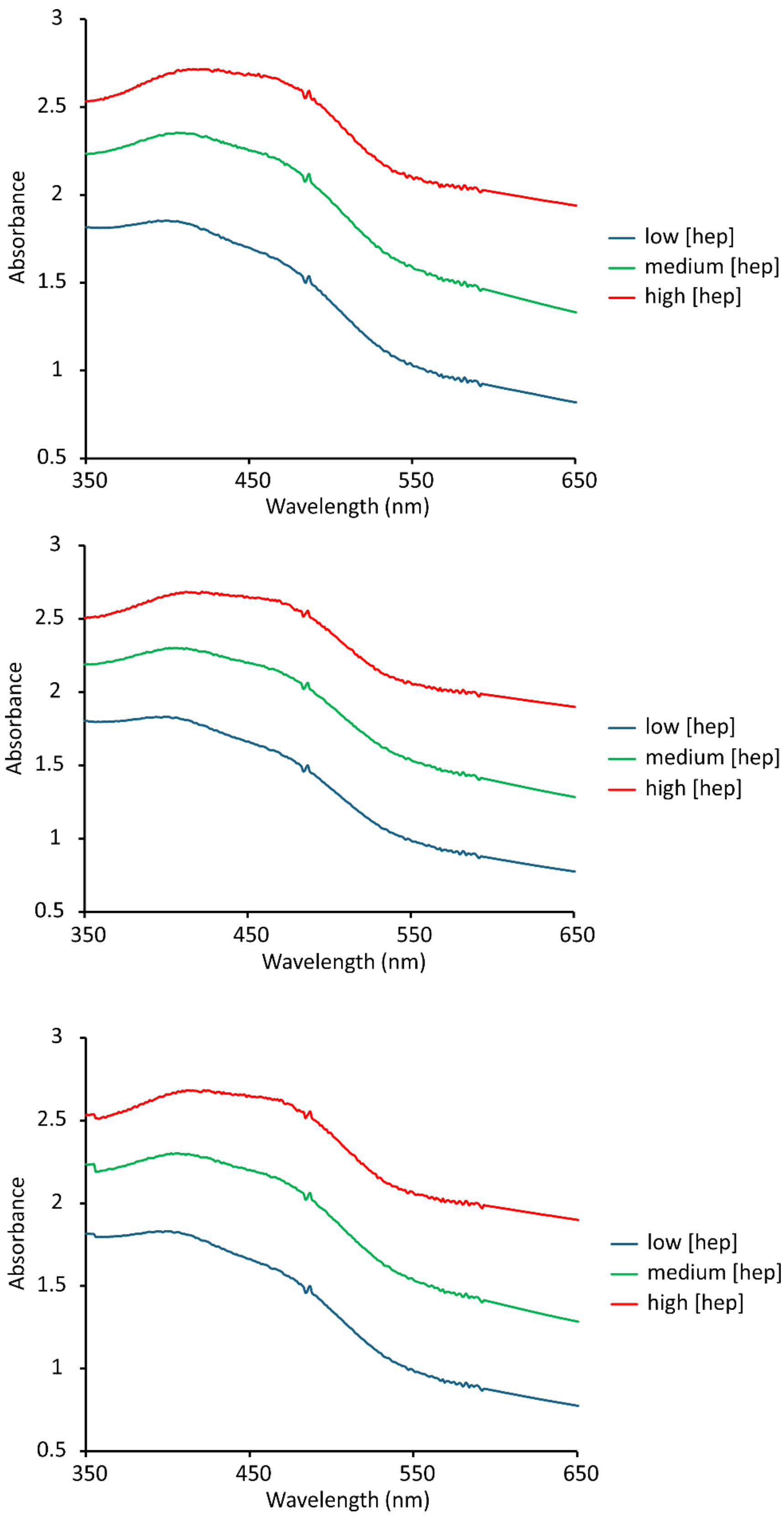

Figure S29. UV absorption spectra showing the spike recovery of the titration of Heparin into a 25:1 mixture of **P12+•MO** in 50% human plasma (pH 7.4)

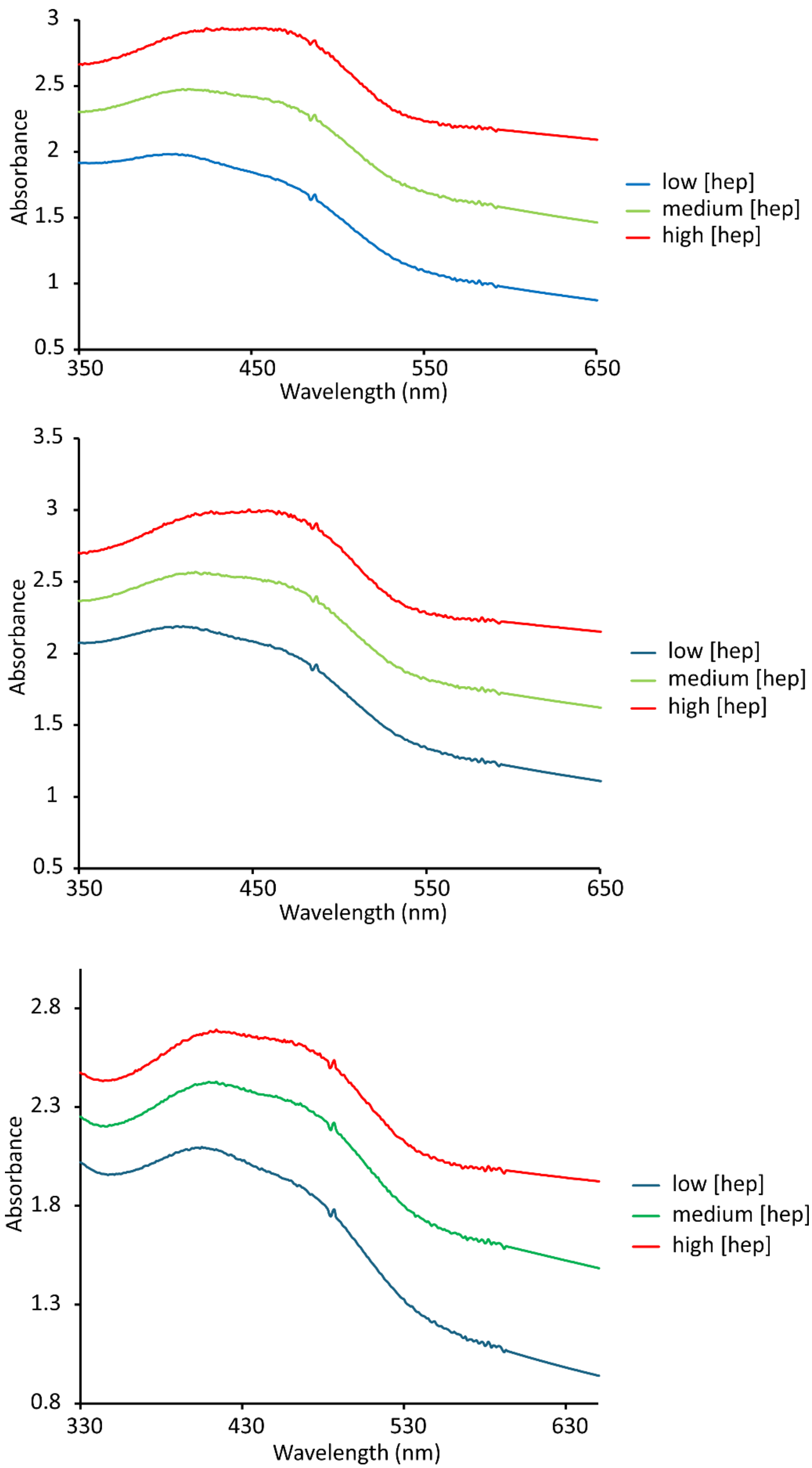

Figure S30. UV absorption spectra showing the spike recovery of the titration of Heparin into a 25:1 mixture of **P12+•MO** in 100% human plasma (pH 7.4)

Table S1: Spike and Recovery Experiment

System	[Heparin]	Run 1	Run 2	Run 3	Average	S.D.
5% Plasma	Low	116.89892	118.817424	99.4591636	111.725169	10.665896
	Medium	112.873404	102.160126	90.6462801	101.89327	11.1159647
	High	115.399589	114.784992	107.952279	112.712287	4.1337253
50% Plasma	Low	80.144641	86.3702271	88.0829791	84.8659491	4.17749238
	Medium	112.273845	104.509269	84.0497328	100.277616	14.5801336
	High	98.9525394	102.712924	103.071833	101.579099	2.28173509
100 % Plasma	Low	111.090614	123.944745	116.582056	117.205805	6.44972617
	Medium	116.602331	91.990207	104.317242	104.30326	12.3060681
	High	99.7463463	121.154547	116.627829	112.509574	11.2826319

UV-Vis Calibration Curves

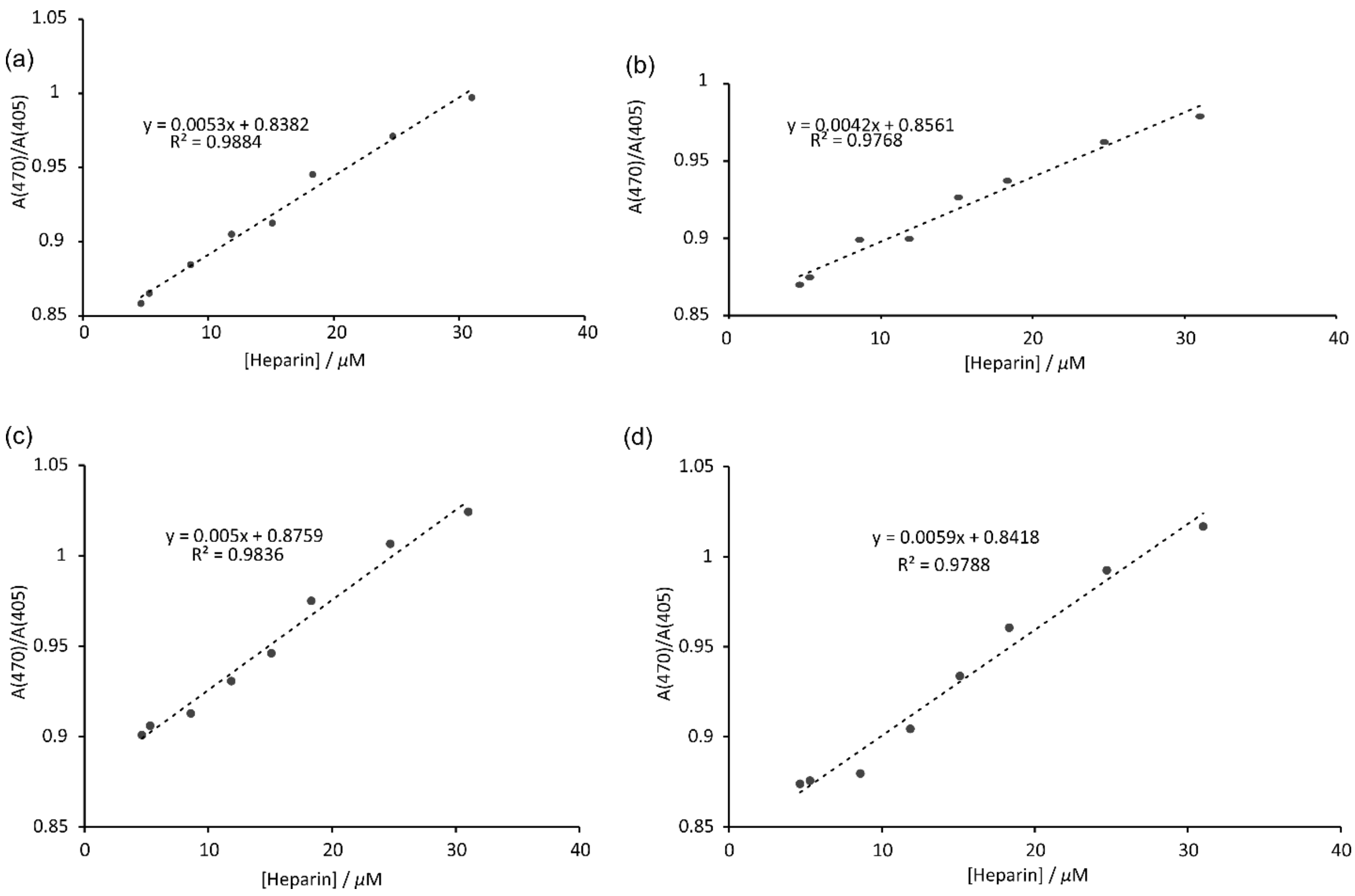

Figure S31: Calibration curve for UV-Vis assay by comparing the absorption maxima of free (470 nm) and bound (405 nm) **MO**, the host-guest concentration: (a) Tris Buffer, (b) 5% Plasma, (c) 50% Plasma, and (d) 100% Plasma.

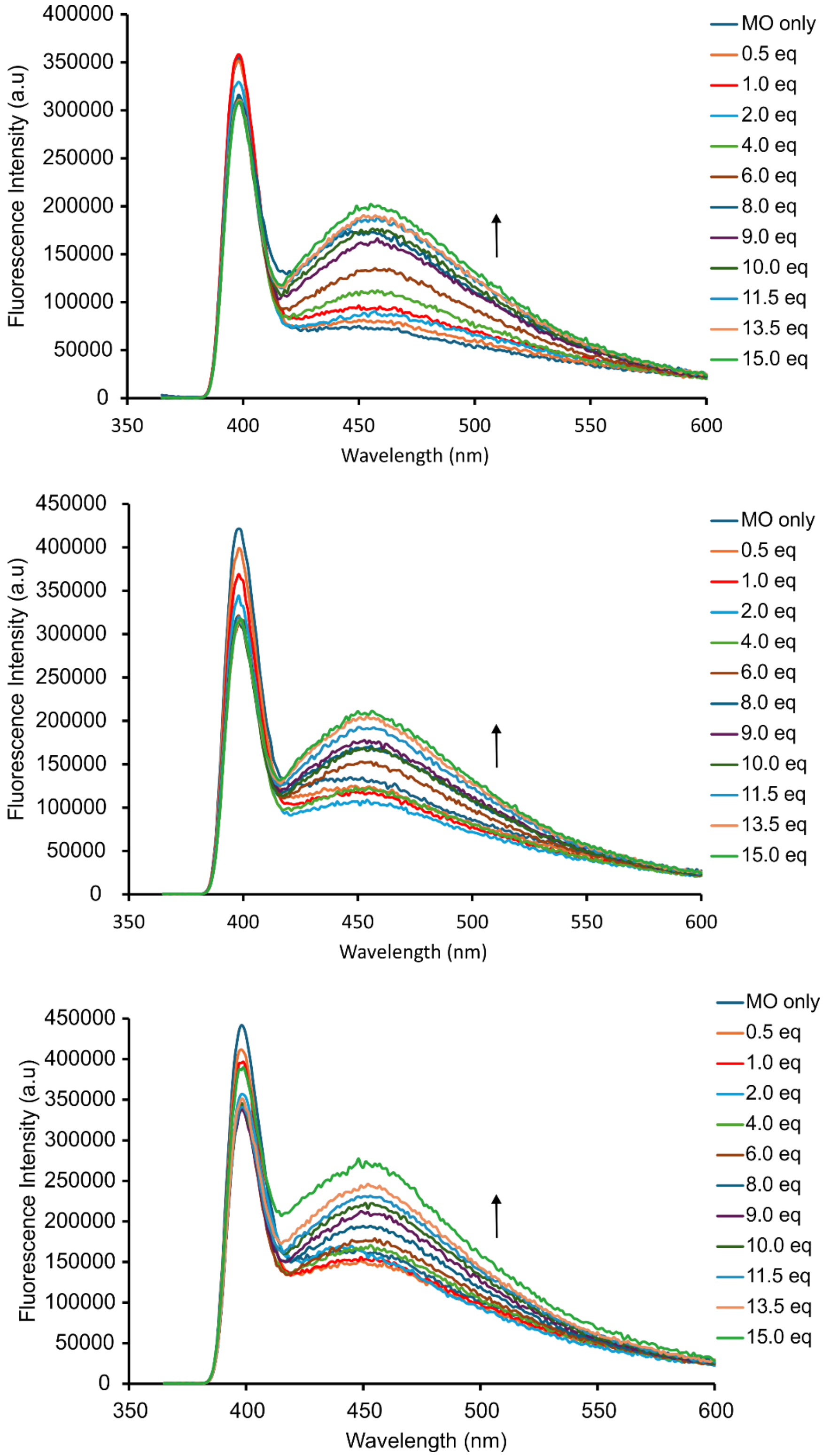

Figure S32. Fluorescence spectra of the titration of **P12+** into **MO** in TRIS-NaCl buffer (10mM Tris, pH 7.4, 150 mM NaCl)

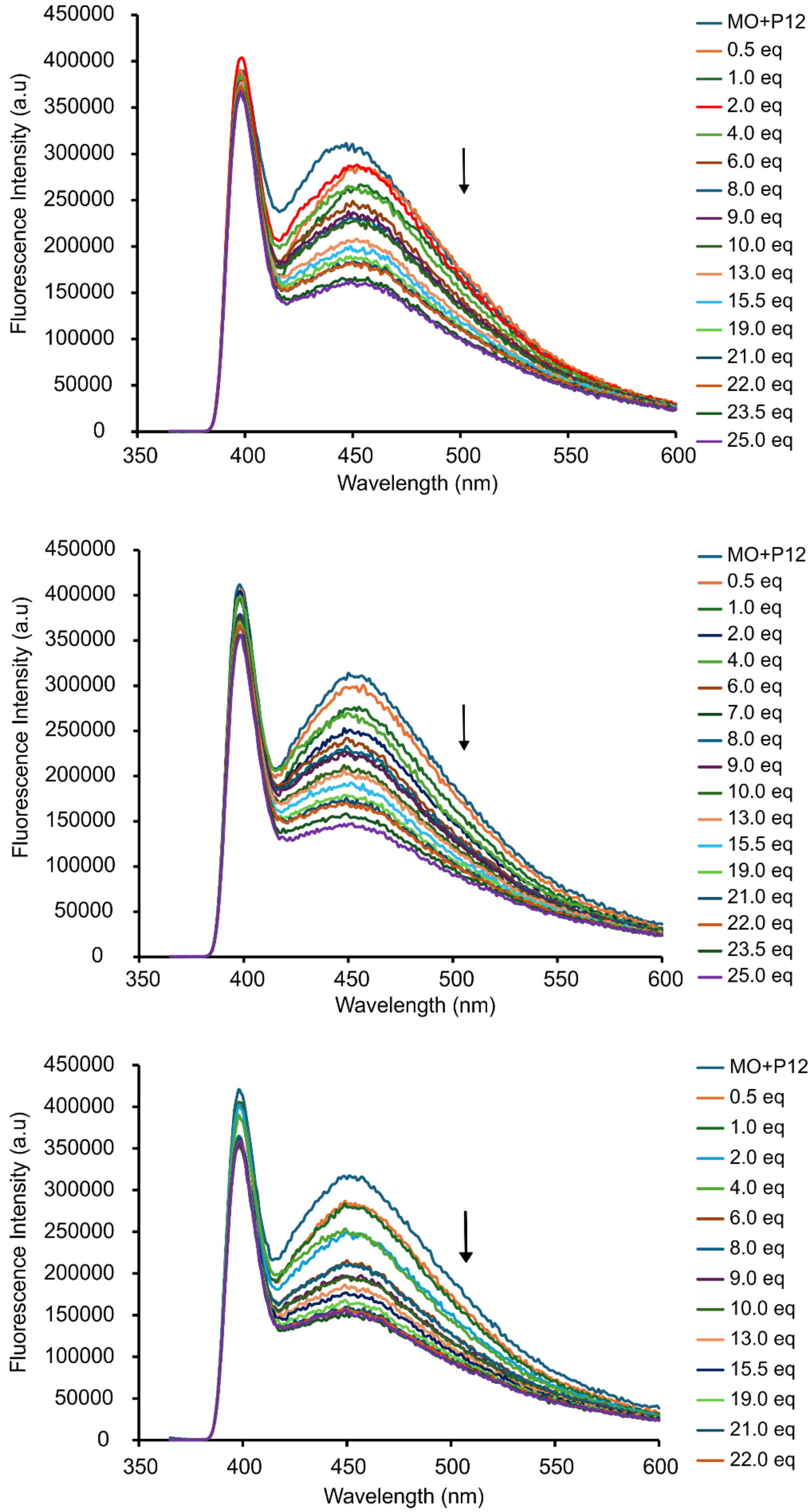

Figure S33. Fluorescence spectra of the titration of Heparin into a 25:1 mixture of **P12+•MO** in TRIS-NaCl buffer (10mM Tris, pH 7.4, 150 mM NaCl).

VIII. Molecular Simulations

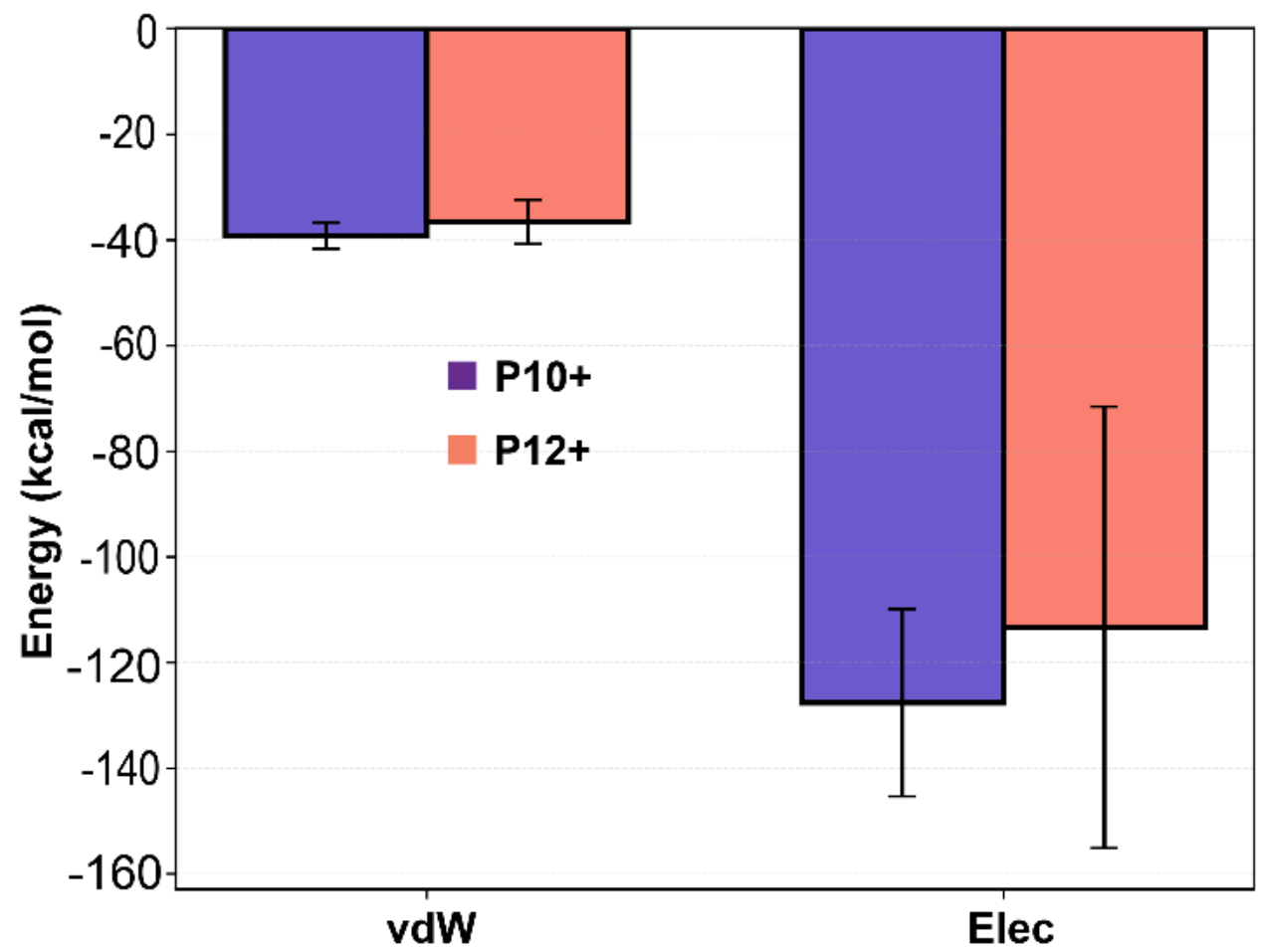

Figure S34. Van der Waals (vdW) and electrostatic (Elec) energies calculated through linear interaction energy (lie) between **MO** and the macrocycles **P10+**/**P12+**.

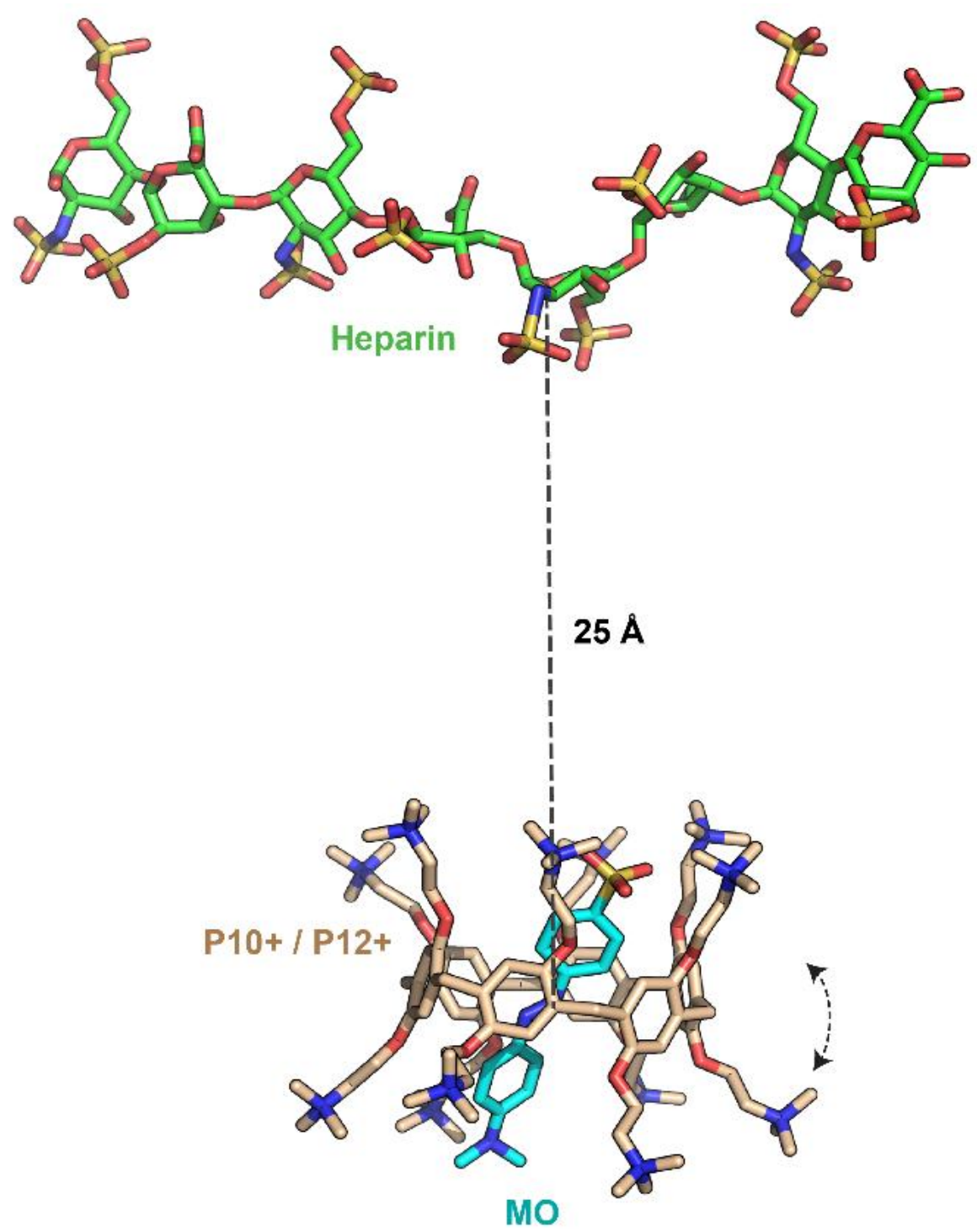

Figure S35. Initial setup of the steered molecular dynamics (SMD) simulations. Heparin was placed 25 Å from the macrocycle COM and pulled toward the COM of the macrocycle. SMD simulations were performed by placing the heparin octamer on both sides of the macrocycle, facing the dimethylamino (–$N(CH_3)_2$) and sulfonate (–SO_3^-) groups of **MO**. The distance restraint was

applied to the center of the macrocycle without restricting its orientation, allowing the macrocycle to have rotational freedom.

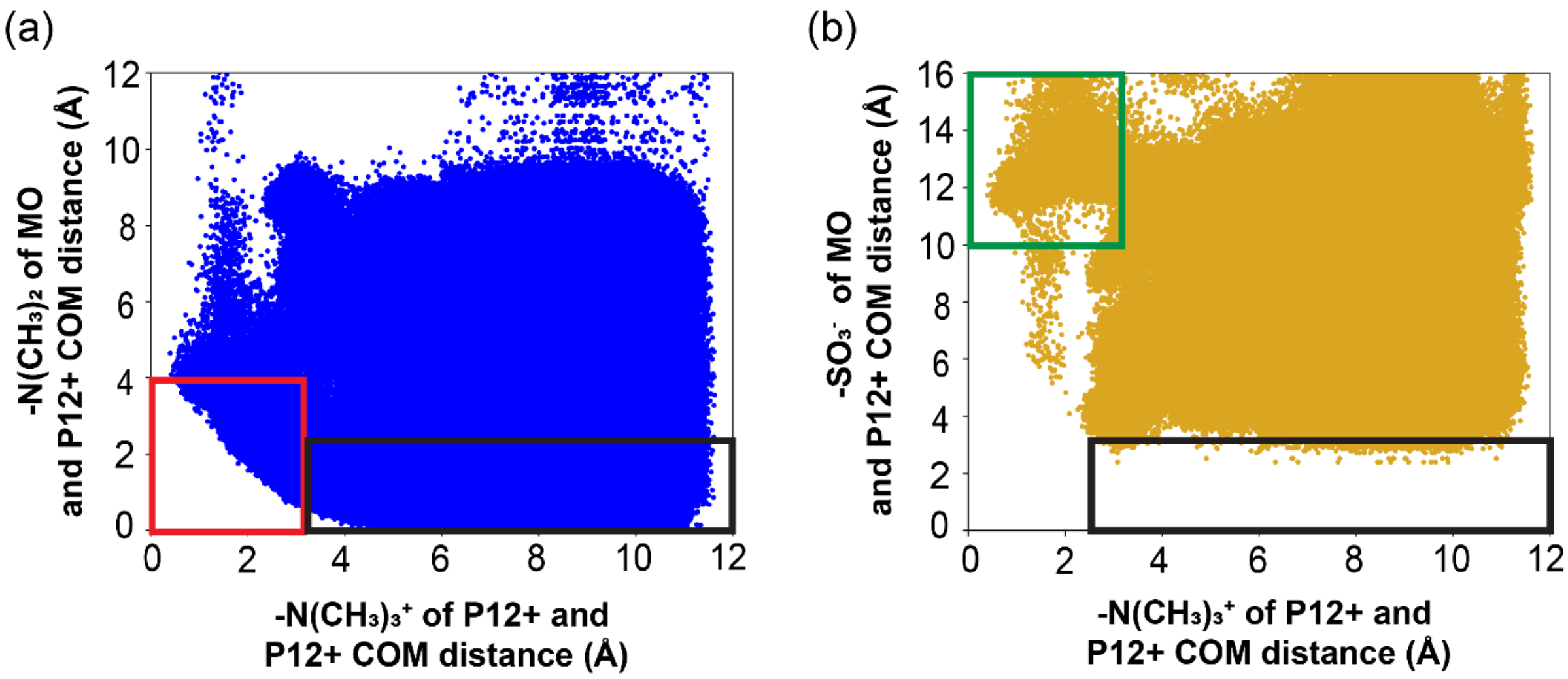

Figure S36. Scatter plots showing the correlation between the dimethylamino ($–N(CH_3)_2$) or sulfonate ($–SO_3^–$) groups of MO and the trimethylammonium ($–N(CH_3)_3^+$) groups of **P12+**. (a) Scatter plot illustrating the correlation between the dimethylamino ($–N(CH_3)_2$) group of MO and the trimethylammonium ($–N(CH_3)_3^+$) groups of **P12+**. The correlation shows that these groups remain separated by distances greater than 3.5 Å, indicating repulsion between them (highlighted in red). (b) Scatter plot illustrating the correlation between the sulfonate ($–SO_3^–$) groups of **MO** and the trimethylammonium ($–N(CH_3)_3^+$) groups of **P12+**. The data indicate that when the trimethylammonium ($–N(CH_3)_3^+$) groups move closer to the macrocycle core, the sulfonate groups are displaced away from the core, suggesting **MO** escape from the macrocycle (highlighted in green). Moreover, the plots shows that only the dimethylamino ($–N(CH_3)_2$) group approaches the macrocycle core, not the sulfonate ($–SO_3^–$) group of **MO** in the simulations (highlighted in black).

VIII. References

1. Montes-García, V., Fernández-Lõpez, C., Gõmez, B., Pérez-Juste, I., García-Río, L., Liz-Marzán, L.M., Pérez-Juste, J., and Pastoriza-Santos, I. (2014) Pillar[5]arene-Mediated Synthesis of Gold Nanoparticles: Size Control and Sensing Capabilities. *Chem. Eur. J*, **20** (27), 8404–8409.
